# Covalent Network Formation Rate Controls Depletion‐Induced Supramolecular Assembly in Hybrid Double Network Hydrogels

**DOI:** 10.1002/anie.8845737

**Published:** 2026-04-11

**Authors:** Mertcan Özel, Sebastian Novosedlik, Tingxian Liu, Lucía López‐Gandul, Heleen Duijs, Hari Veera Prasad Thelu, Ciqing Tong, Roxanne E. Kieltyka

**Affiliations:** ^1^ Department of Supramolecular and Biomaterials Chemistry, Leiden Institute of Chemistry Leiden University Leiden The Netherlands

**Keywords:** double networks, hydrogels, macromolecular crowding, squaramides, supramolecular materials

## Abstract

The introduction of a secondary covalent polymer network is a powerful approach to extend the usable application range of supramolecular hydrogels. While it is recognized that dramatic changes in mechanics can occur with their addition, there is a lack of insight into the impact of added covalent polymers on hydrogels with underlying supramolecular filament nanostructures. Here we show that through controlling the rate of covalent network formation by the inverse electron‐demand Diels–Alder reaction, the mesoscale architecture of the supramolecular network can be programmed. Slow macromonomer crosslinking enables depletion‐induced supramolecular assembly of the supramolecular filaments into bundles above a critical macromonomer concentration, whereas rapid covalent network formation halts this dynamic process by effectively locking in the low‐nm scale supramolecular nanostructures. This kinetic difference further translates into mechanically distinct hydrogels, where slow‐forming hybrid networks reveal a two‐fold increase in toughness as compared to fast‐crosslinked networks, thanks to the bundled supramolecular filaments. Through harnessing the macromolecular crowding capacity of reactive macromonomers and their reaction kinetics, a new axis to control the hierarchical structure of supramolecular hydrogels through depletion forces is unlocked that can be exploited to shape this soft matter class for numerous applications.

## Graphical abstract

Tuning the rate of covalent network formation in hybrid double network hydrogels regulates whether supramolecular filaments retain their low‐nm structures or depletion‐induced supramolecular assembly takes place forming bundles. This kinetic handle programs the mesoscale architecture and toughness of the hydrogels, establishing a link between macromonomer reaction rate and macromolecular crowding, unlocking a design strategy for hierarchical engineering of soft materials.

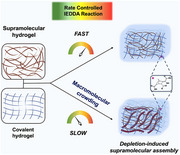

## 1 Introduction

Supramolecular hydrogels based on reversible, noncovalent interactions are a rapidly evolving class of soft materials with numerous potential applications, namely in the biomedical field [1, 2]. The noncovalent character of the materials, built up from the association of molecular recognition groups on covalent polymers or small molecules that self‐assemble into filamentous nanostructures, endows them with dynamic features such as stimuli‐responsiveness or self‐healing, yet they are often mechanically weak. To extend their mechanical range, secondary covalent polymer networks can be added that enable the supramolecular hydrogels to sustain much greater tensile and compressive loads and deformation before failure [3, 4, 5, 6, 7, 8, 9, 10, 11]. The concentration of local stresses is reduced due to the efficient energy dissipation mechanisms of the hybrid hydrogels [12]. While it is known that the concentrations of the individual networks and their crosslinking are critical parameters to modulate their mechanics, the impact of applied covalent polymers particularly on filamentous supramolecular networks is often overlooked, but can be a critical handle to further refine their properties.

Within such hybrid soft materials, a sufficient amount of macromonomers is needed to form a space‐spanning secondary covalent network. At the same time, it is well known from the fields of protein science, (bio)polymers, and nanomaterials that increasing polymer concentrations can give rise to crowding effects, steering the formation of higher order structures through depletion forces [13, 14, 15, 16, 17, 18, 19, 20]. The macromolecular crowding concept has been recently brought over to the field of supramolecular polymers [21, 22, 23, 24, 25]. In these few examples, inert covalent polymers of varied molecular weight and concentration are relied on to guide the formation of hierarchical filament assemblies largely in organic solvents. This concept remains to be deployed in the gel phase, where it could have major consequences on gel architecture and mechanics that are commonly targeted properties in soft matter.

Secondary covalent networks can be prepared within supramolecular hydrogels using reactive (macro)monomers with various chemistries. Depending on the selected chemistry, the reaction rate can be tuned from fast to slow, or even modulated by various stimuli (e.g., pH, light). One example of a reaction with fine rate tunability is the inverse electron‐demand Diels–Alder (IEDDA) [26, 27, 28, 29, 30, 31, 32, 33]. This reaction has been extensively used for bioconjugation, including crosslinking of hydrogels due to its bioorthogonality and operation under physiological conditions [34, 35, 36, 37, 38]. The IEDDA reaction involves a cycloaddition between an electron‐deficient diene (e.g., tetrazine) and an electron‐rich dienophile (e.g., norbornene). The reaction rate is strongly influenced by the electronic properties of the reactant pair, particularly the attached functional groups that modulate their HOMO–LUMO energy gap [39, 40]. This chemical ligation strategy has been exploited on covalent polymers such as polyethylene glycol (PEG), gelatin, alginate and cellulose to control the rate of hydrogelation from minutes [38] to hours [41, 42], and the mechanics of the materials. However, this kinetically tunable bioconjugation chemistry has yet to be used to prepare interpenetrated hydrogels but can be an attractive handle to modulate the rate of formation of the independent polymer networks.

**FIGURE 1:**
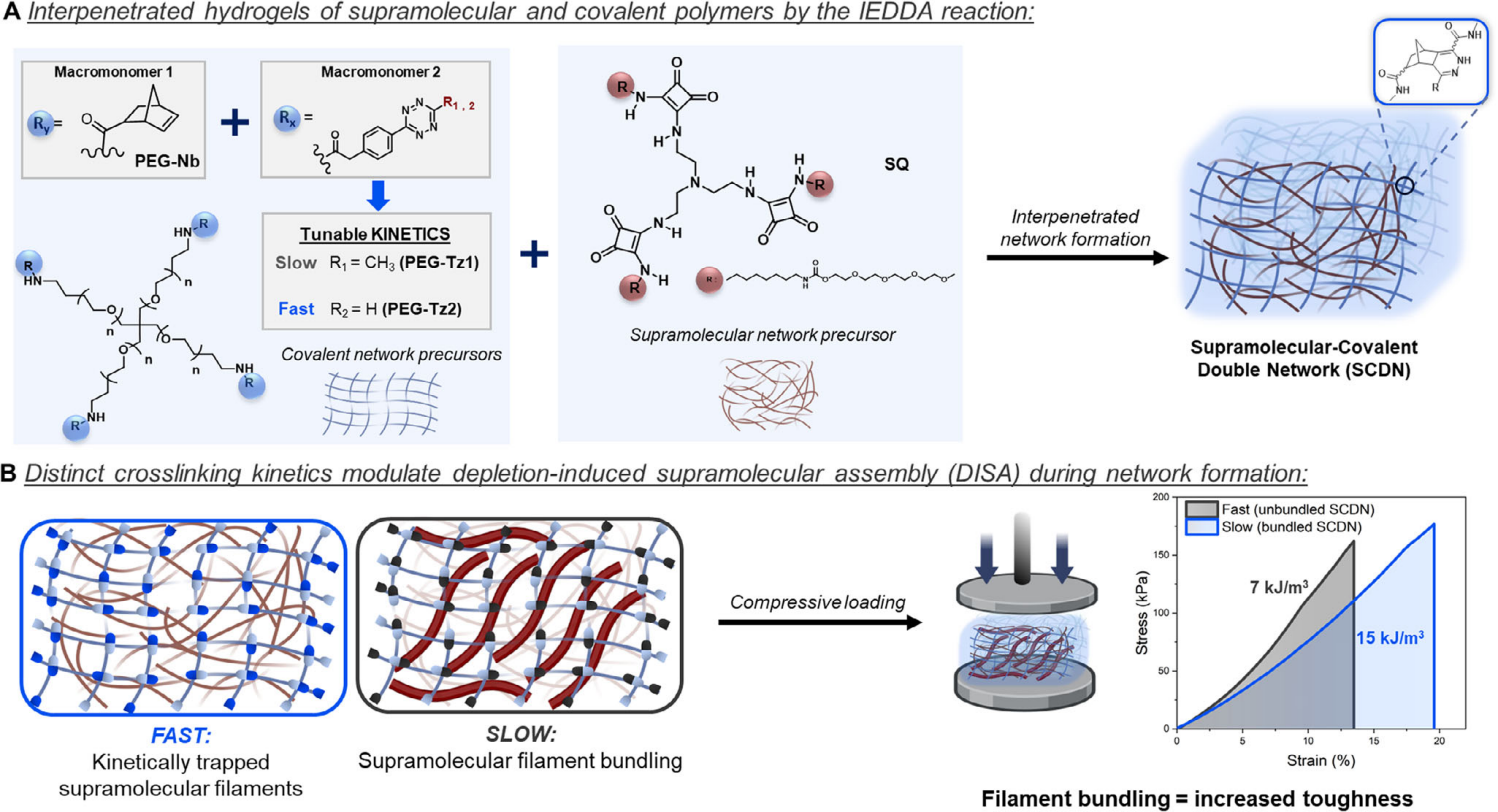
Schematic representation of the supramolecular and covalent double network (SCDN) hydrogels prepared in this work. (A) Macromonomers end‐functionalized with tetrazines bearing different substituents at the C‐6 position (**PEG–Tz1** and **PEG–Tz2**) control the electronic character and rate of the inverse electron‐demand Diels–Alder (IEDDA) click reaction with PEG–norbornene (**PEG–Nb**) to obtain covalent networks. These covalent networks form in the presence of the squaramide‐based tripodal amphiphiles (**SQ**) that self‐assemble into supramolecular filaments and together they compose the SCDN hydrogel. (B) Conceptual illustration of the SCDNs (blue network) formed by covalent network formation at different rates with supramolecular filaments (brown network), and the effect of depletion‐induced supramolecular assembly (DISA) on the compressive behavior of the hybrid double network hydrogel. Dark red filaments represent the supramolecular bundles formed by brown‐colored individual supramolecular nanostructures.

We herein examine the impact of controlling the rate of covalent network formation on the structure and function of hybrid supramolecular and covalent double network hydrogels (SCDN). By systematically varying the macromonomer crosslinking rate using IEDDA reaction pairs with differing reaction rates based on tetrazine (**PEG‐Tz1** or **PEG‐Tz2**) and norbornene (**PEG–Nb**) end groups, we explore for the first time how the kinetics of covalent network formation of 4‐arm PEG macromonomers influences the filament architecture of squaramide (**SQ**)‐based supramolecular polymers and the mechanics of the resulting hybrid double network hydrogels (Figure 1). Our results reveal that slow covalent network formation promotes filament bundling through depletion‐induced supramolecular assembly (DISA), whereas fast network formation hampers this dynamic process, locking in the low‐nm scale supramolecular filaments during the hydrogelation process. The dramatically altered microstructure of the bundled SCDNs leads to enhanced mechanical properties, especially under compressive loads, that can be harnessed for numerous soft matter applications.

## 2 Results and Discussion

We first selected two different tetrazine moieties to control the rate of network formation with norbornene through the IEDDA reaction on 4‐arm PEG polymers. We chose 1,2,4,5‐tetrazines with methyl and hydrogen substituents at the C‐6 position based on their differences in electronic character that lead to distinct IEDDA reaction rates. Second order reaction rate constants for methyl and hydrogen C‐6 substituted tetrazines with trans‐cyclooctene (TCO) in PBS solutions have been reported within 210 and 26 000 M^−1^ s^−1^, respectively [43]. Because of the greater electron‐donating character of the methyl substituent, we expected a slower rate of covalent network formation due to an increase in the HOMO–LUMO energy gap between tetrazine and norbornene. Consequently, we coupled methyl(Tz1)‐ and hydrogen(Tz2)‐substituted tetrazines, through their respective NHS esters to a 4‐arm PEG amine (*M*_w_ = 10 kDa), obtaining both macromonomers (**PEG–Tz1** and **PEG–Tz2**) with high degrees of end‐functionalization (≥85%) (Supporting Information 1.2). Using a similar approach, we also prepared the norbornene analogue (**PEG–Nb**) needed to perform the IEDDA click reaction.

We quantified the difference in rate of covalent network formation using the tetrazines of distinct electronic character by oscillatory rheology. We prepared 4 mM **PEG–Tz1/Nb** and **PEG‐Tz2/Nb** hydrogels by mixing solutions of the two macromonomers in phosphate‐buffered saline (PBS, pH 7.4) at an equivalent concentration according to the protocol outlined in the Supporting Information (Supporting Information 1.1.1 and Scheme S1A). Time sweep measurements of the single networks (SN) at 37°C displayed that **PEG–Tz1** reacts slowly with **PEG–Nb** (gel point ca. 30 min for a 4 mM gel) while **PEG–Tz2** reacts rapidly (ca. 2 min for a 4 mM gel) at the same total polymer concentration (Figure S1A). Because of the significant difference in SN crosslinking rates, we could also use gel inversion to visually monitor the gelation processes (Figure S2). The final storage moduli, *G*′, of the SN hydrogels depended both on the macromolecular concentration and crosslinking kinetics of the IEDDA reaction pair. At a total macromonomer concentration of 4 mM, the slower network **PEG–Tz1/Nb** reaches a plateau *G*′ in the range of 3 kPa, whereas the fast network **PEG–Tz2/Nb** attains 5 kPa. Increasing the macromonomer concentration further to 6 mM, promotes a parallel increase of *G*′ up to 6 and 10 kPa, respectively (Figure S1A). These results demonstrate that the faster IEDDA crosslinking chemistry of **PEG–Tz2** yields stiffer single covalent networks, consistent with earlier reports using the same reaction pairs [44, 45].

Achieving pronounced differences in the rate of covalent hydrogel formation of the SNs through the electronically distinct tetrazines, we proceeded to incorporate supramolecular filaments based on a tripodal **SQ** that yields gel phase materials in water [46, 47, 48]. Because the IEDDA crosslinking chemistry takes place soon after mixing both **PEG–Tz1** or **PEG–Tz2** and **PEG–Nb** components, we devised a mixing protocol to accommodate their rapid gelation, especially for combinations with **PEG–Tz2** (Supporting Information 1.1.1 and Scheme S1B). Briefly, we prepared films of **SQ** from organic solvents, rehydrated them in PBS and sonicated them at 4°C for 20 min. We subsequently added the dispersed supramolecular filaments to the solid PEG macromonomers required for hydrogelation, **PEG–Tz1** or **PEG‐Tz2** and **PEG‐Nb**. To prevent premature hydrogelation, we kept the **SQ** solutions on an ice bath prior to mixing with the PEG macromonomers. We combined both networks at the desired concentrations, particularly above the concentration where the single covalent networks form gels, that is, at least 1.5 mM **PEG–Tz1** or **PEG–Tz2** and 1.5 mM **PEG–Nb**.

**FIGURE 2:**
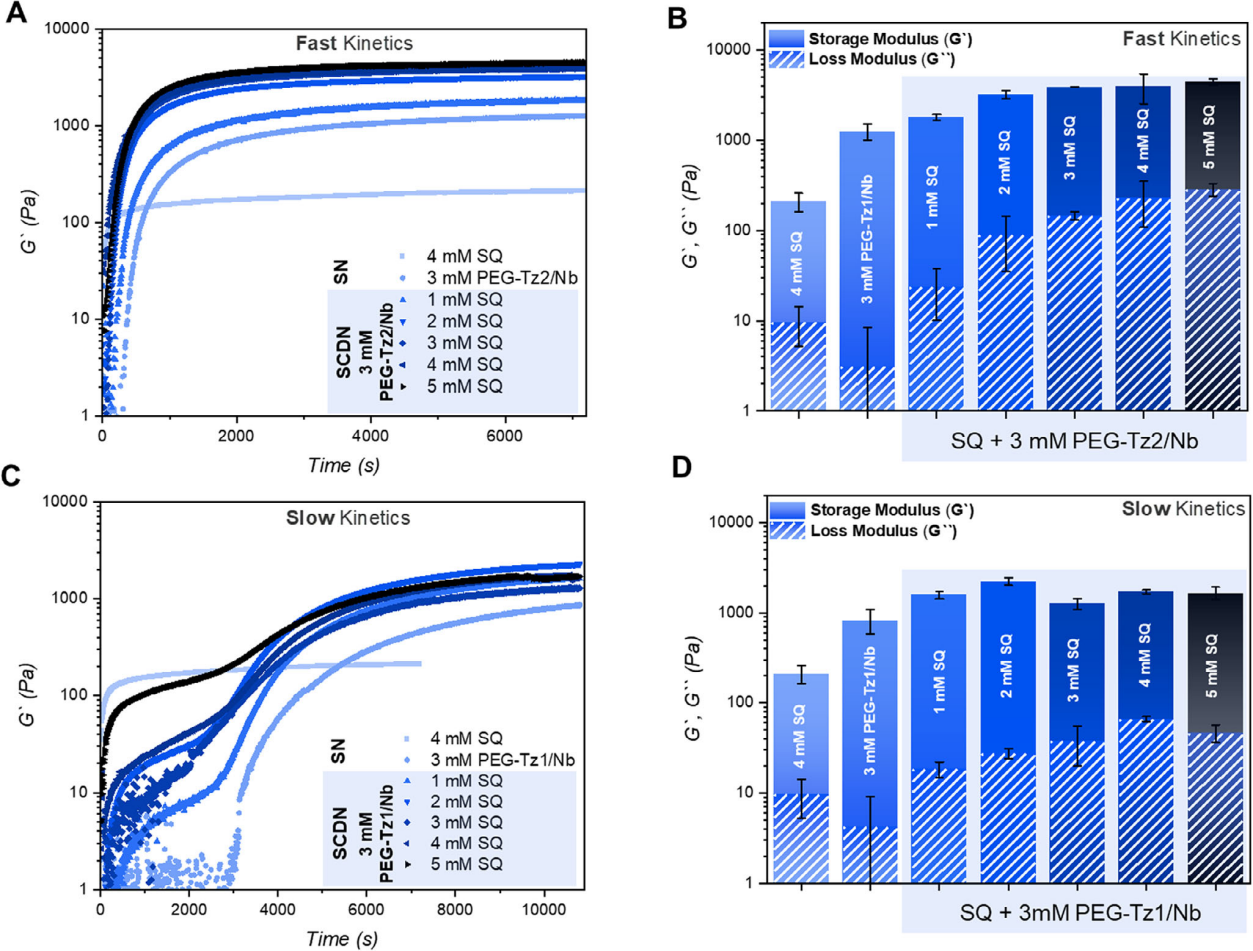
Oscillatory rheology measurements of SCDN hydrogels using tetrazines of distinct electronic character. Storage modulus (*G*′) evolution over time for hydrogels containing increasing concentration of **SQ**, prepared using either fast **PEG–Tz2** (A,B) or slow **PEG–Tz1** (C,D) macromonomers with **PEG–Nb**. Plateau *G*′ (solid bars) and *G*′′ (striped bars) values for various **SQ** concentrations in fast **PEG–Tz2** (B) and slow **PEG–Tz1** (D) SCDNs. Time sweep measurements were performed using 0.05% strain at 1 Hz and 37°C. Mean ± SD, based on *n* = 3.

We carried out oscillatory rheology time sweep measurements to elucidate the impact of adding the supramolecular network on the SCDN crosslinking rate and stiffness. The rate of crosslinking of **PEG–Tz1** or **PEG–Tz2** with **PEG–Nb** is retained as in the SNs, even with the addition of the supramolecular filaments (Figure 2 and S1B). The time sweep profiles also point to the significant contribution of the covalent networks to the final plateau *G*′ of the SCDNs. Looking further into the evolution of the profiles over time, we observe a marked two‐stage gelation profile for the slow **PEG–Tz1/Nb** whereas the faster **PEG–Tz2/Nb** displayed only a single stage (Figure S1B). Hence, the delayed crosslinking of the **PEG–Tz1/Nb** network indicates that mechanical changes in the supramolecular network occur in tandem with the formation of the covalent network.

We further examined the consequence of increasing the **SQ** concentration (1–5 mM) in SCDN hydrogels with 3 mM **PEG–Tz1/Nb** or **PEG–Tz2/Nb**. For the faster reacting **PEG–Tz2/Nb** that shows a profile with a single stage, raising the **SQ** concentration resulted in a consistent increase in plateau *G*′ values that level off around ≈ 4–5 kPa within 60 min at concentrations above 2 mM (Figure 2A,B). In contrast, the slower reacting **PEG–Tz1/Nb** displayed an initial increase in *G*′ to about 100 Pa corresponding to development of the **SQ** network, followed by a gradual rise to a plateau G′ of ca. 1–2 kPa after the covalent PEG network formed over the course of 3 h (Figure 2C,D). For **PEG–Tz1/Nb**, we recorded a subtle decrease in *G*′ with increasing **SQ** concentration above 3 mM, hinting at a distinct structural evolution within the SCDN due to the slower rate of covalent network formation. Despite the distinct gelation kinetics of the fast and slow SCDN hydrogels their final *G*′ values are similar in the low kPa range, indicating that both systems ultimately form stiff interpenetrated networks once the crosslinking reaction is complete, with the covalent network contributing significantly to their mechanics.

As entangled supramolecular filaments provide opportunities to access hydrogels with complex mechanical characteristics, we probed the consequence of the rate of covalent network formation on the stress relaxation behavior of the SCDNs. We measured the stress relaxation of the hydrogels (1–5 mM **SQ** and 3 mM **PEG–Tz1/Nb** or **PEG–Tz2/Nb**) under constant strain (10%) at 37°C (Figure S3). For both **PEG–Tz1/Nb** and **PEG–Tz2/Nb**, the rate and extent of stress relaxation with increasing **SQ** concentration roughly falls within the same range from the lowest to the highest tested concentrations. However, significant differences can be observed in the stress relaxation trends over the various **SQ** monomer concentrations for the fast and slow covalent networks. The faster **PEG–Tz2/Nb** network relaxes stress in a more monotonic and reproducible manner as the **SQ** concentration is increased, whereas the slower **PEG–Tz1/Nb** lacks this trend and exhibits a more irregular increase in stress relaxation. These results generally show that increasing the supramolecular filament concentration promotes stress relaxation in the SCDNs, whereas the covalent network dominates the measured relaxation responses as observed in the time sweep measurements. Moreover, frequency sweeps (0.1–10 Hz) of the independent networks and the SCDNs (3 mM **SQ** and 3 mM **PEG–Tz2/Nb**) corroborate the contribution of the supramolecular network to the observed stress relaxation behavior and support the recorded slow relaxation dynamics (Figure S1C). We further applied the Kohlrausch–Williams–Watts (KWW) equation (Supporting Information Equation 1) [49] to determine the stress‐relaxation half‐times (*t*_1/2_), characteristic time constant (*τ*_k_) and β that represents the distribution of relaxation time scales for the various conditions (Figure S4 and Table S1). The fast crosslinked **PEG–Tz2/Nb** exhibits a modest increase in the rate and extent of stress relaxation when compared against the slower **PEG–Tz1/Nb** at comparable compositions. The *t*_1/2_ values further decrease for both networks with increasing supramolecular content, encompassing a broad spectrum of relaxation time scales extending from ≈ 2.5 h to ≈ 3 days, comparable to other reported dynamic hydrogels [50, 51]. The *β* values for the hybrid networks range from 0.35 for the fast **PEG–Tz2/Nb** network to 0.25 for the slower **PEG–Tz1/Nb** at a 4 mM concentration of **SQ**. The *β* values of both fast and slow networks that are significantly less than 1 point to multiple relaxation modes and heterogeneity of the hydrogels, due to their structural complexity being composed of covalent and supramolecular components.

**FIGURE 3:**
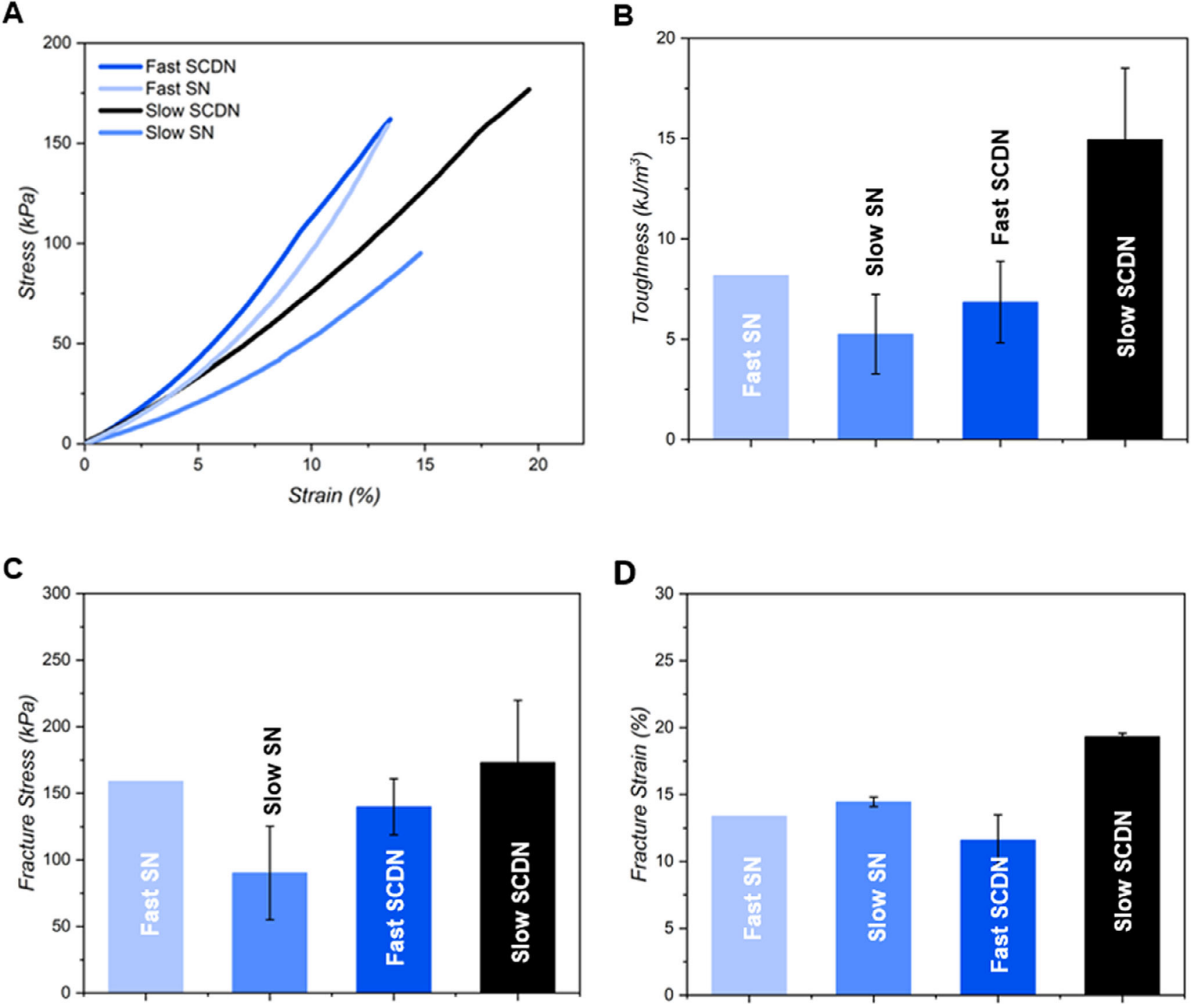
Compressive rheology results of the covalent single network (SN) and SCDN hydrogels. (A) Uniaxial stress–strain curves, (B) toughness, (C) fracture stress, and (D) fracture strain. Concentration: SN: 3 mM **PEG–Tz1** **/Nb** (slow) or **PEG–Tz2/Nb** (fast), SCDN: 4 mM **SQ** and 3 mM **PEG Tz1/Nb** (slow) or **PEG–Tz2/Nb** (fast). The samples were compressed at a speed of 10 µm/s. Mean ± SD, based on *n* = 2.

As the addition of a secondary covalent network fortifies the supramolecular network substantially in shear tests, we became interested in understanding if the different rates of covalent network formation promote distinct mechanical behavior in compression. We executed uniaxial compression tests on both fast and slow SN and SCDNs (4 mM **SQ** and 3 mM **PEG–Tz1/Nb** or **PEG–Tz2/Nb**) (Figure 3). The stress–strain profiles reveal that slow **PEG–Tz1/Nb** SCDNs exhibit a markedly higher fracture stress, strain, and toughness compared to the fast **PEG–Tz2/Nb** (Figure 3A). Quantitatively, we found an increase in toughness from ~7 kJ m^−3^ for the fast **PEG–Tz2/Nb** to ∼15 kJ m^−3^ for the slow **PEG–Tz1/Nb** SCDNs (Figure 3B), accompanied by higher fracture stress and strain (Figure 3C,D). The striking improvement in the compressive properties of the slow **PEG–Tz1/Nb** SCDN further indicates a difference in the hybrid hydrogel microstructure that enables efficient load transfer and energy dissipation.

**FIGURE 4:**
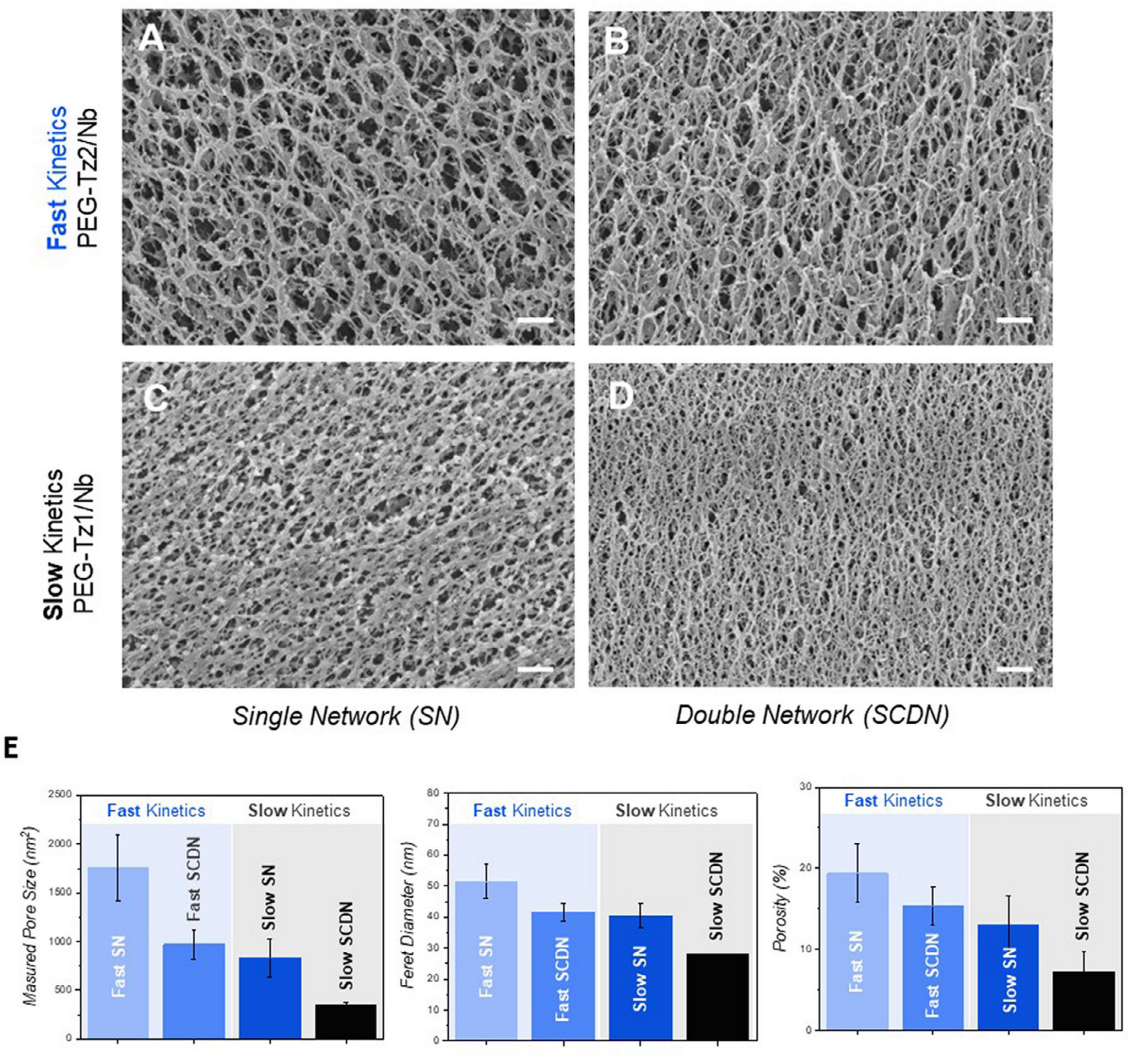
Cryogenic scanning electron microscopy (cryo‐SEM) imaging of SN (*left*) and SCDN (*right*) hydrogels (A‐D). Micrographs of fast **PEG‐Tz2/Nb** (A,B) and slow **PEG‐Tz1/Nb** (C,D). (E) Calculated pore parameters based on the cryo‐SEM images. Concentration: SN: 3 mM **PEG–Tz1/Nb** or **PEG–Tz2/Nb**, SCDN: 4 mM **SQ** and 3 mM **PEG–Tz1/Nb** or **PEG–Tz2/Nb**. Scale bar: 300 nm.

Given the clear differences in rheological behavior of the hybrid hydrogels in shear and compression, we further examined their microstructures by cryo‐scanning electron microscopy (cryo‐SEM). Both hybrid hydrogels show microstructures dominated by their respective covalent SNs formed at different crosslinking rates. The faster crosslinking **PEG–Tz2/Nb** shows qualitatively larger pore sizes as compared to **PEG–Tz1/Nb** at the same total macromonomer concentration. This observation highlights that the difference in the rate of formation of the covalent networks promotes visibly distinct hydrogel microstructures (Figure 4A,C). These microstructural differences were largely conserved when adding the supramolecular filaments. However, the covalent networks were indistinguishable from the supramolecular filaments precluding visualization of their impact by this imaging method (Figures 4B,D and S5, and Table S2).

**FIGURE 5:**
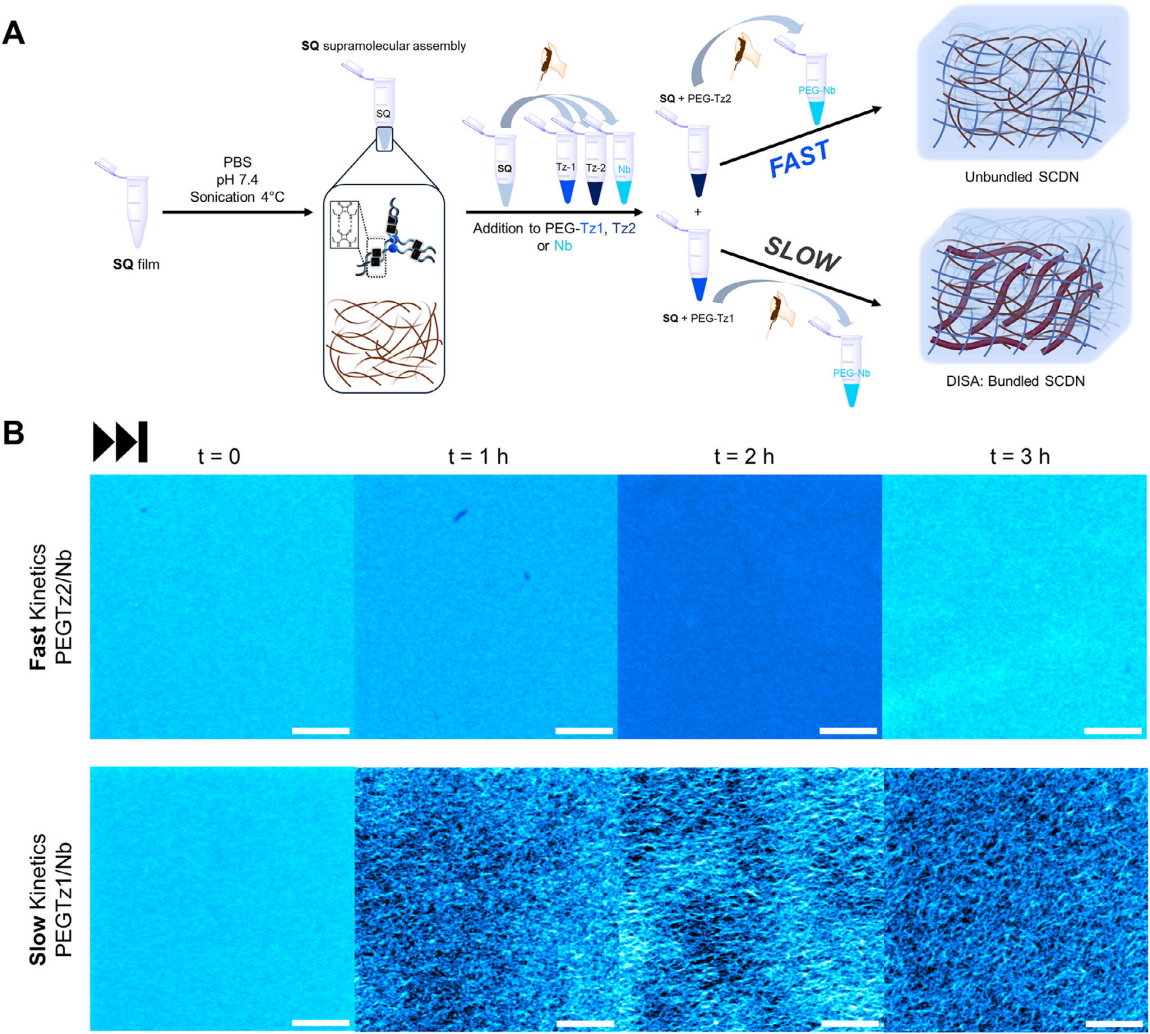
(A) Schematic representation of preparation protocols for fast and slow SCDN hydrogels. (B) Representative confocal imaging time series of fast (*top*) and slow (*bottom*) SCDN hydrogels (4 mM **SQ** and 3 mM **PEG–Tz1/Nb** or **PEG–Tz2/Nb**) from *t* = 0 to 3 h in time intervals of 1 h. Brightness and contrast were adjusted in the images. Raw (unprocessed) micrographs are provided in Figure S6. **SQ** hydrogels contained 1 mol% **SQ‐Cy5**. Scale bar: 10 µm.

We subsequently collected confocal laser scanning microscopy (CLSM) images to gain insight into the presentation of supramolecular **SQ** filament network in the SCDN hydrogels. To enable their visualization, we co‐assembled a fluorescently labelled **SQ** monomer functionalized with a sulfo‐Cyanine5 dye (1 mol % **SQ–Cy5**) and its native **SQ** counterpart, prior to preparation of the SCDNs (4 mM **SQ** and 3 mM **PEG–Tz1/Nb** or **PEG‐Tz2/Nb**) (Figure 5A). Initially, we observed a uniform fluorescence signal consistent with the low nm‐scale diameters of the supramolecular filaments (∼5 nm [47]) lying below the diffraction limit.

Further imaging over time by CLSM, we unveiled dramatic differences in the supramolecular filaments with respect to the two rates of IEDDA crosslinking (Figure 5B). In the slow crosslinking **PEG–Tz1/Nb** network (Figure 5B bottom), the **SQ** filaments gradually (*t* = 0–3 h) thicken and elongate, yielding bundled supramolecular assemblies hundreds of nanometers in diameter and several microns in length. These dynamic architectural changes of the supramolecular filaments largely appeared by the 1h imaging time point and evolved over the course of the next 2 h in line with the time to reach a plateau in *G*′ in the oscillatory rheology time sweeps. Quantitative analysis of confocal cross‐sections reveals average bundle thicknesses of 296 ± 40 nm at *t* = 3 h (Figure S7). Conversely, the **SQ** filaments in the fast‐crosslinking **PEG–Tz2/Nb** network (Figure 5B top and Figure S6) remain homogeneously dispersed without any detectable bundle formation, even after monitoring for a period of 1 week. Hence, the fast rate of covalent network formation of **PEG–Tz2/Nb** kinetically traps the filaments before lateral interactions start to occur. The observed temporal modulation of the supramolecular network architecture suggests that the rate of covalent network formation dictates whether the filaments remain dynamic or become arrested from undergoing hierarchical association.

**FIGURE 6:**
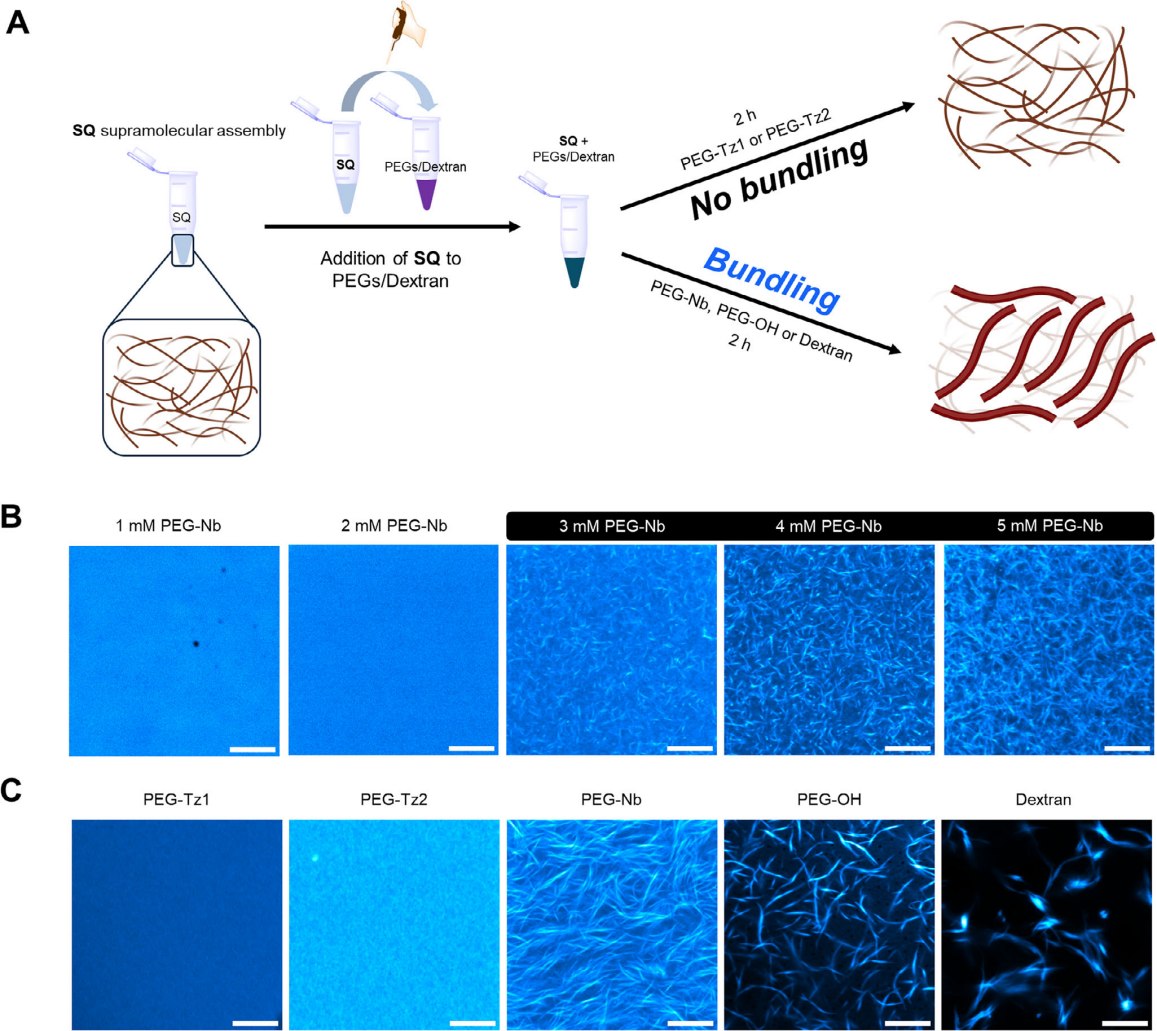
(A) Schematic representation of preparation protocols for supramolecular **SQ** hydrogels (4 mM) with various macromolecular crowders. (B) Confocal images of **SQ** hydrogels with **PEG–Nb** (1–5 mM), imaged immediately after mixing (*t* = 0 h). (C) Confocal images of **SQ** hydrogels with different macromolecular crowders (3 mM PEGs and 0.4 mM dextran), imaged after 3 h with incubation at 37°C. **SQ** hydrogels contained 1 mol% **SQ‐Cy5**. Scale bar: 10 µm.

We followed the evolution of the **SQ** filaments in the presence of the individual reactive macromonomers, **PEG–Tz1**, **PEG–Tz2,** and **PEG–Nb**, to deconvolute the origin of the observed architectural changes in **SQ** during covalent network formation by the IEDDA reaction (Figure 6A–C). Addition of the various macromonomers at final concentration of 3 mM for **PEG–Tz1**, **PEG–Tz2**, and **PEG–Nb**, yielded distinct results on the **SQ** filaments (Figure 6C). Interestingly, **PEG–Nb** gave rise to bundles with diameters in the range of 287 ± 52 nm (Figure S7), while **PEG–Tz1** and **PEG–Tz2** retained the dispersed low‐nm scale filaments. These results hint that the reactive PEG‐based macromonomers at the concentrations used to prepare the SCDNs can act as macromolecular crowders that encourage the hierarchical assembly of the **SQ** filaments through depletion forces.

We imaged a concentration series of **PEG–Nb** (1–5 mM) to determine the threshold of macromonomer needed to obtain bundling of the supramolecular filaments (Figure 6B). Through these experiments, we established that the critical concentration lies in the range of 3 mM, in line with excluded‐volume mechanisms requiring sufficient polymer concentration to exert depletion forces that promote filament bundling [52]. In the absence (Figure S8) or at low concentrations of **PEG–Nb** (≤ 2 mM), the supramolecular network remains as dispersed low‐nm filaments below the diffraction limit. Upon increasing the concentration to 3 mM, visible bundles began to emerge, indicating that the threshold concentration for bundling was surpassed. We also labeled **PEG‐Nb** at this concentration with a fluorescent dye (AF 488 Tetrazine, 5‐isomer) to understand its presentation in the **SQ** hydrogels, observing a uniform fluorescent signal with no detectable domains (Figure S9). At higher **PEG‐Nb** concentrations (4–5 mM), the bundled filaments became more interconnected through the excluded volume effect, leading to additional supramolecular organization of the noncovalent network (Figure 6B).

Given the differences in bundling of the supramolecular filaments by the various PEG‐based macromonomers, we evaluated whether the observed dynamic behavior is connected to their end groups (Figure 6C). We first examined a 4‐arm PEG‐OH to probe whether the terminal group chemistry influences the crowding efficiency observed between **PEG–Tz1 or PEG–Tz2** and **PEG–Nb**. Similar to **PEG–Nb**, PEG–OH effectively induces fibril bundling with an average bundle diameter of 328 ± 59 nm, indicating that PEG macromonomers with other end groups can also facilitate this process. We additionally applied dextran (70 kDa), a commonly used macromolecular crowder [13, 53] with a distinct chemical structure, to evaluate its potential to bundle the supramolecular filaments. In the presence of dextran at 0.4 mM, pronounced **SQ** bundles were observed with an average diameter of 448 ± 62 nm, larger than those formed using the PEG‐based crowders (Figure S7). Together, these results demonstrate that polymers of distinct chemical composition and architecture—whether PEG or dextran—can induce supramolecular filament undling purely through entropic excluded‐volume effects.

To rationalize the disparate bundling behavior induced by macromolecular crowding using the various reactive PEG macromonomers, we evaluated their dispersions by dynamic light scattering (DLS). We analyzed 4‐arm PEG macromonomers (*M*_w_ = 10 kDa) bearing different terminal groups at a concentration of 3 mM. The unmodified PEG–OH exhibited low scattering intensity with a stable and average derived count rate of ∼500 kcps. In contrast, both **PEG–Tz1** and **PEG–Tz2** displayed multimodal scattering profiles, indicating the presence of multiple species with spontaneous aggregation, showing mean derived count rates that were 53‐fold and 27‐fold greater, respectively. The **PEG–Nb** macromonomer remained more homogeneously dispersed, yielding only a 5‐fold greater count rate. These results demonstrate that tetrazine‐functionalized PEGs exhibit a pronounced tendency to aggregate in aqueous solution, whereas **PEG–Nb** and PEG–OH remain colloidally stable under identical conditions. The larger size of **PEG–Tz1** and **PEG–Tz2** likely diminishes their effective crowding capacity, as smaller and more homogeneously distributed crowders, such as **PEG–Nb**, exert stronger depletion forces due to their higher accessible surface area and greater effective volume exclusion [54]. Overall, these studies point out that excluded‐volume effects alone are sufficient to promote supramolecular assembly of the **SQ** filaments, yet the ability of DISA to manifest within the SCDN hydrogels ultimately depends on the rate of covalent network formation that modulates the temporal window of filament mobility before the network becomes kinetically arrested.

**FIGURE 7:**
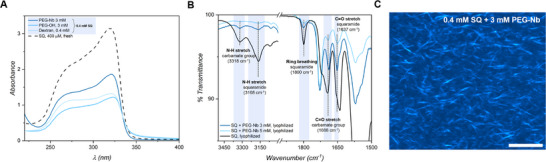
(A) UV–vis spectra of 0.4 mM **SQ** (black dotted line) and 0.4 mM **SQ** with different macromonomers: 3 mM **PEG–Nb** (darkest blue), 3 mM PEG‐OH (blue), and 0.4 mM dextran (lightest blue). (B) FT‐IR spectra of lyophilized samples of 4 mM **SQ** and **PEG–Nb** mixtures (3 mM, blue, and 5 mM, black) highlighting the hydrogen‐bonding regions. (C) CLSM image of mixture of 0.4 mM **SQ** and 3 mM **PEG–Nb**, showing the formation of bundles. Scale bar: 10 µm.

We next performed spectroscopic experiments to provide a view into the molecular packing of the supramolecular monomers in the bundled filaments (Figures 7A–C). Through UV–vis spectroscopy, we observed that 0.4 mM **SQ** solutions in the presence of the different macromolecular crowders, **PEG–Nb**, PEG‐OH and dextran, show identical features to those obtained in the absence of crowder that display the characteristic band pattern for the head‐to‐tail hydrogen‐bonded assembly of **SQs** (*λ*_max_ = 318 nm and a shoulder at 266 nm, Figure 7A) [47]. However, the absorbance of the bundled **SQ** samples is approximately halved for all macromonomers as compared to that registered for solely **SQ**, which is on par with the formation of larger assemblies [55]. We confirmed that bundling of the **SQ** filaments takes place at the UV‐Vis concentrations by CLSM, where we image similar morphologies to those obtained for the more concentrated samples (Figures 7C and S10). We also conducted FT‐IR experiments to probe the molecular packing at the higher concentrations used in CLSM experiments (4 mM **SQ**, Figure 7B). We recorded the spectra of lyophilized samples of **SQ** in the absence and presence of **PEG‐Nb**. The FT‐IR spectra show indistinguishable features in the NH stretching bands of **SQ** and carbamate, ring breathing of **SQ** and C═O stretching bands of carbamate in the presence of the **PEG–Nb** macromonomer (Figures 7B and S11A). The C═O stretching bands of **SQ** overlap with those afforded by the macromonomer and thus could not be used in the analysis (Figure S11B). These results point to an identical aggregation pattern of the **SQ** monomers in presence and in absence of the polymers and further support that depletion forces guide dynamic lateral association of the **SQ** filaments into their bundled form. Collectively, these experiments highlight that DISA can be exploited to tune the properties of supramolecular materials through modulating their mesoscale assemblies while retaining monomer packing at the nanoscale that may be essential to preserve their function.

## 3 Conclusion

This study demonstrates that the kinetics of covalent network formation plays a decisive role in controlling the hierarchical assembly of the supramolecular filaments in hybrid supramolecular and covalent double network hydrogels. Modulating the reaction rate of the macromonomers, we uncover for the first time a relationship between macromonomer reactivity and macromolecular crowding on the supramolecular filament architecture. This work is distinct from earlier approaches involving supramolecular polymers that rely on the use of inert crowders. Particularly, slow network formation paves the way for mobility of the supramolecular nanostructures permitting DISA to occur whereas rapid crosslinking kinetically traps the system at earlier assembly stages, retaining low‐nm scale filaments. Mechanically, this kinetic distinction translates into modular stress‐relaxation and compressive properties. Fast‐forming double networks display faster stress relaxation but lower toughness, consistent with their low‐nm scale supramolecular filaments. On the other hand, slow‐forming double networks exhibit two‐fold enhancement in toughness, consistent with their bundled filament architectures that can effectively dissipate compressive stress through reversible supramolecular interactions.

Excitingly, this work provides a conceptual framework to engineer the architecture and mechanics of supramolecular soft matter by harnessing the dynamic nature of supramolecular materials and kinetic nature of covalent networks. However, this strategy that involves regulating macromolecular crowding through reactive macromonomers can be further extended to other classes of nanomaterials beyond those that are supramolecular and open possibilities to explore this concept in the diverse application space where macromolecular crowders are applied. We envisage the design principles disclosed here, in combination with the large diversity of covalent bond reactions that operate under numerous conditions (e.g., pH, temperature, light) and available supramolecular monomers, will provide a new axis to construct hierarchically programmable soft matter at the mesoscale, tuning features that are essential for their use in the biomedical domain but also for emerging technologies in flexible electronics, soft actuators and responsive (bio)interfaces.

## Conflicts of Interest

The authors declare no conflicts of interest.

## Supporting information



The authors have cited additional references within the Supporting Information [56, 57, 58, 59, 60].
**Supporting File**: anie71968‐sup‐0001‐SuppMat.docx.

## Data Availability

The data that supports the findings of this study are available in the Supporting Information of this article.
