# Supplementary material for: Covalent Network Formation Rate Controls Depletion‐Induced Supramolecular Assembly in Hybrid Double Network Hydrogels
Source: Angew Chem Int Ed Engl. 2026 Apr 11;65(21):e8845737. doi: 10.1002/anie.8845737 (PMC13182229; doi:10.1002/anie.8845737)
Supplement: Supplementary file 1 — The authors have cited additional references within the Supporting Information [56, 57, 58, 59, 60]. Supporting File: anie71968‐sup‐0001‐SuppMat.docx. [file ANIE-65-e8845737-s001.docx]

***Supporting Information for***

**Covalent Network Formation Rate Controls Depletion-induced Supramolecular Assembly in Hybrid Double Network Hydrogels**

**Authors:** Mertcan Özel, Sebastian Novosedlik, Tingxian Liu, Lucía López-Gandul, Heleen Duijs, Hari Veera Prasad Thelu, Ciqing Tong, Roxanne E. Kieltyka^*^

**Affiliations:** Department of Supramolecular and Biomaterials Chemistry Leiden Institute of Chemistry P.O. Box 9502, 2300 RA, The Netherlands ^*^E-mail: r.e.kieltyka@chem.leidenuniv.nl

Table of Contents

[1. Experimental Procedures 3](#_Toc225665775)

[1.1 Materials and Methods 3](#_Toc225665776)

[1.1.1. Hydrogel preparation 3](#_Toc225665777)

[1.1.2. UV-Vis spectroscopy 5](#_Toc225665778)

[1.1.3. Fourier transform infrared (FTIR) spectroscopy 5](#_Toc225665779)

[1.1.4. Oscillatory Rheology 5](#_Toc225665780)

[1.1.5. Cryogenic Scanning Electron Microscopy (cryo-SEM) 6](#_Toc225665781)

[1.1.6. Confocal Laser Scanning Microscopy (CLSM) 7](#_Toc225665782)

[1.1.7. Dynamic Light Scattering (DLS) 8](#_Toc225665783)

[1.2 Synthetic Procedures 8](#_Toc225665784)

[1.2.1 4-arm PEG Functionalization (PEG-Tz1, PEG-Tz2 and PEG-Nb) 8](#_Toc225665785)

[1.2.2. Synthetic route of the sulfo-Cyanine5 dye Functionalized SQ Monomer (SQ-Cy5) 10](#_Toc225665786)

[1.2.3. ^1^H-NMR and ^13^C-NMR Spectra 13](#_Toc225665787)

[1.2.4. LC-MS Analysis 28](#_Toc225665788)

[2. Additional Figures and Tables 29](#_Toc225665789)

[3. References 38](#_Toc225665790)

# 1. Experimental Procedures

## **1.1 Materials and Methods**

**Materials.** Tetra-arm hydroxy-terminated PEG (M_w_:10 kDa) was obtained from Creative PEGWorks. Amine-terminated PEG (M_w_:10 kDa) and PEG (M_w_:10 kDa) were obtained from Jenkem Technology. Sulfo-Cy5-NHS ester and AF 488 Tetrazine (5-Isomer) were purchased from Lumiprobe. Deuterated dimethyl sulfoxide (DMSO-d_6_) and chloroform (CDCl_3_) were procured from Eurisotop. Anhydrous dimethylformamide (DMF) and tetrahydrofuran (THF) were dried over 3 or 4 Å molecular sieves. Dulbecco’s phosphate buffered saline (PBS) was obtained from Sigma Aldrich. All other chemicals and reagents for synthesis were purchased from Sigma Aldrich and used without further purification.

**Purification.** HPLC purification of the squaramide-based supramolecular monomer was executed on a setup equipped with C18 column. A gradient from 1-10% CH_3_CN/H_2_O with 0.1% trifluoroacetic acid (TFA) over 15 min at a flow rate of 12 mL/min was used.

**NMR spectroscopy.** ^1^H and ^13^C NMR spectra were acquired on a Bruker DMX-400 and Bruker DPX-300 MHz at 298K. Chemical shifts (δ) are relative to the solvent used and given in parts per million (ppm).

**Mass spectroscopy analysis.** LC-MS analysis was performed on a TSQ Quantum Access MAX system equipped with a Gemini 3 µm C18 110 Å 50×4.60 mm column (UV detection at 214 nm and 254 nm, mass detection range: 160 to 3000 (Da)). The mobile phase consisted of a gradient of 10-90% of H_2_O-CH_3_CN with 0.1% trifluoroacetic acid (TFA) over 13.5 minutes.

### 1.1.1. Hydrogel preparation

***Single network (SN) covalent PEG hydrogels:*** PEG macromonomers (**PEG-Tz1** or **PEG-Tz2**, and **PEG-Nb**) were dissolved separately in the required amount of phosphate-buffered saline (PBS, pH 7.4) to achieve final gel concentrations ranging from 2 to 6 mM. Specifically, for the preparation of 3 mM gels, **PEG-Tz1** or **PEG-Tz2** (1.8 mg) and **PEG-Nb** (1.8 mg) were each dissolved in PBS (60 µL) and then mixed to initiate gel formation (Figure S1A), yielding **PEG-Tz1/Nb** or **PEG-Tz2/Nb** hydrogels, respectively.

***SQ hydrogels:*** The supramolecular hydrogels were prepared following a previously reported protocol with some modifications.^1^ Briefly, thin films were generated at the desired final concentrations (e.g., 1–5 mM) from a 6 mM stock solution of **SQ** in DMSO under a continuous nitrogen flow overnight using a Smart Evaporator (BioChromato). The resulting thin films were then rehydrated with the appropriate volume of PBS, vortexed, and subjected to sonication in an ultrasonic bath at 4 °C for 20 minutes to obtain homogeneous solutions. To prevent premature self-assembly and gelation, **SQ** solutions were stored in an ice bath prior to mixing with the covalent PEG network components.

***Hybrid supramolecular and covalent double network (SCDN) hydrogels:*** Hybrid double network hydrogels were prepared by adding the PEG macromonomers directly into freshly prepared **SQ** solutions in PBS on ice, using the same protocol described above. Briefly, to prepare 4 mM **SQ** and 3 mM **PEG-Tz1/Nb** or **PEG-Tz2/Nb** hydrogels, an aliquot of the 6 mM **SQ** stock solution in DMSO (120 µL) was transferred into an LC vial and the solvent was evaporated overnight under a nitrogen stream to yield the SQ thin film. Next, PBS (180 µL) was added to the film, followed by vortexing for 60 seconds and sonication at 4 °C for 20 minutes. Separately, PEG macromonomers **PEG-Tz1** or **PEG-Tz2** (2.0 mg) and **PEG-Nb** (2.0 mg) were weighed in Eppendorf tubes and dissolved in the **SQ** solution (67 µL) individually by vortex. The supramolecular and covalent double network (SCDN) hydrogels were subsequently prepared by adding the **PEG-Tz1+SQ** or **PEG-Tz2+SQ** solution on top of the **PEG-Nb+SQ** solution and pipetting up and down 10 times to obtain homogeneous gels before any further experiments (Figure S1B).


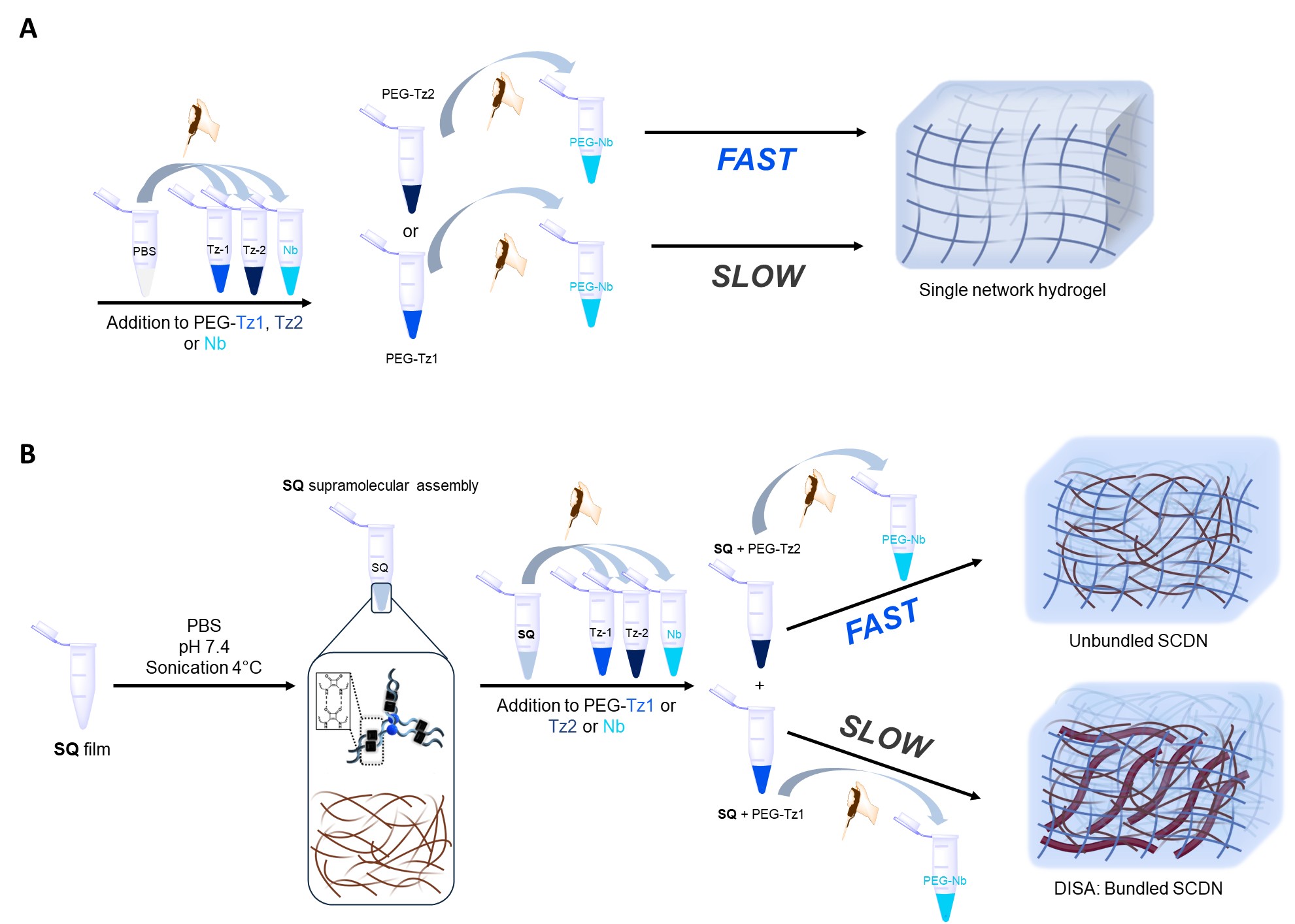


**Scheme S1.** (A) Preparation of SN hydrogels from **PEG-Tz1** or **PEG-Tz2** and **PEG-Nb**. (B) Preparation of **SQ** and SCDN hydrogels.

### 1.1.2. UV-Vis spectroscopy

UV-Vis absorption spectra were acquired using a Cary 300 UV-Vis spectrophotometer. All measurements were performed scanning from 200 to 450 nm using a quartz cuvette with a path length of 1 mm. ***Sample preparation:*** 0.4 mM **SQ** samples for UV-Vis experiments were prepared by rehydrating a thin film prepared from DMSO taken from a 6 mM stock solution. The solutions in water were then sonicated for 20 min at 4 ^o^C and added to the pre-weighed **PEG-Nb** to reach the desired macromonomer concentration (3 mM) and further left to stand for 3 h to permit the bundling process. After this period, the solution was introduced in a 1 mm pathlength cuvette, and a UV-Vis spectrum was recorded at 20 ^o^C.

### 1.1.3. Fourier transform infrared (FTIR) spectroscopy

FT-IR spectra in the solid state were recorded on a Perkin Elmer Spectrum Two Spectrophotometer with MIR detector and KBr windows with a resolution of 4 cm^-1^. ***Sample Preparation:*** The procedure is analogous to that described previously for UV-Vis sample preparation. However, the samples were prepared at a 4 mM **SQ** concentration for FT-IR. After the 3 h stabilization period, the samples were lyophilized to obtain a white powder that was subsequently used for ATR measurements.

### 1.1.4. Oscillatory Rheology

Oscillatory rheology shear and compression tests were carried out on a Discovery HR-2 hybrid rheometer using a parallel plate geometry (20 mm diameter, 1.995°) at 37 ± 0.2 °C. with a Peltier temperature controller and a solvent trap filled with MQ water to avoid evaporation and drying during the measurements. For oscillatory rheology measurements, the prepared hydrogel (103 µL) was pipetted onto the bottom plate, and the geometry was lowered to a gap distance of 300 µm.

***Shear tests:*** Time sweep measurements were executed at a frequency of 1 Hz and strain of 0.05% The measurements were repeated for at least three independent replicates. The average plateau modulus was calculated by taking the mathematical average of the last 10 minutes of collected data. After a plateau was reached in the storage modulus during the time sweep, stress relaxation measurements were executed at 10% strain for 7200 s. The stress was normalized to the average stress during the first five seconds of the measurement. Normalized data was imported into MATLAB and fitted to a stretched exponential Kohlrausch-Williams-Watts equation (equation S1). The normalized stress σ/σ_0_ was modeled as an exponential decay modulated by the characteristic time constant τ_k_ and the stretching parameter β describing the distribution of relaxation timescales. A β value of 1 represents a single relaxation mode, while values closer to 0 are indicative of a heterogeneous distribution of relaxation modes.^2^ To quantify the rate of stress relaxation for the various dynamic hydrogels, t_1/2_, defined as the time at which the stress reached half of its initial value during the measurement, was determined from the fitted data using MathWorks-MATLAB.

$\frac{\sigma}{\sigma_{0}}=e^{{-\left( \frac{t}{\tau_{k}} \right)}^{\beta}}$ (**Equation S1**)

***Compression tests:*** The time-sweep experiments at 37 °C performed over the course of 2 and 3 hours for fast and slow network hydrogels, respectively. Then, an axial compression test was conducted compressing the sample to 80% of its initial height (600 µm to 120 µm) at a compression rate of 10 µm/s at 37 °C. Toughness values of hydrogels were calculated by integrating the area under the compressive stress-strain curves using OriginPro software.

1.1.5. Cryogenic Scanning Electron Microscopy (cryo-SEM)

***Sample preparation:*** Hydrogel samples were first sectioned into 90 μm-thick slices using a Leica vibratome. These slices were then trimmed into smaller fragments suitable for high-pressure freezing (HPF) carriers (Ø 3 mm, depth 2 × 50 μm; Art. 390, Wohlwend). All manipulations were performed in phosphate-buffered saline (PBS) to maintain hydration. Each carrier was filled with PBS, and the hydrogel specimen was placed between two opposing carriers, which were subsequently closed and vitrified using a high-pressure freezer (CryoCapCell, Paris, France).

Following vitrification, the frozen samples within their carriers were loaded to a cryo-holder to transfer to the Quorom preparation chamber (PP3010T, Quorum Technologies, Laughton, UK) for sublimation and platinum coating. Sublimation was conducted at −90 °C for 7 minutes to remove surface ice, followed by sputter coating with platinum at 5.0 mA for 30 seconds to ensure surface conductivity. The coated samples were then mounted onto a cryo-holder and transferred into the chamber of a Zeiss Crossbeam 550 FIB/SEM system (Carl Zeiss Microscopy GmbH, Oberkochen, Germany). Imaging was performed under cryogenic conditions, with the sample stage maintained at −140 °C and the anticontaminator at −175 °C to prevent contamination. Secondary electron (SESI) and InLens detectors were employed for image acquisition at an accelerating voltage of 2 kV and a beam current of 50 pA to minimize electron beam-induced damage. Hydrogel orientation and directionality analysis were performed the Directionality plug-in in the Fiji software. This plug-in executes a fast Fourier transform of the pictures (Fourier components mode) to retrieve the angle coverage.

1.1.6. Confocal Laser Scanning Microscopy (CLSM)

CLSM imaging was performed using a Leica Stellaris 8 equipped with a Leica HC PL APO CS2 63× oil immersion objective (NA 1.40). Image resolution, scanning speed, detector gain, and laser intensity were optimized individually for each sample. Scale bars were added using OMERO software.

***CLSM sample preparation and imaging:*** **SQ** hydrogels were co-assembled with 1 mol% **SQ-Cy5**, following the same procedure as standard **SQ** gel formation. SCDN hydrogels were formed by dissolving PEG macromonomers (**PEG-Tz1** or **PEG-Tz2** and **PEG-Nb**) directly into freshly prepared **SQ** solutions in PBS. For 3 mM gels, each PEG component (1.8 mg) was dissolved in **SQ** solution (60 µL). The two components were then mixed to initiate gelation. For kinetic studies (fast and slow network formation), **PEG-Tz1** or **PEG-Tz2** (30 µL) and **PEG-Nb** (30 µL) were pre-mixed in an Eppendorf tube. An aliquot (50 µL) was transferred to an Ibidi µ-Slide 18 Well Glass Bottom slide. For 37 °C conditions, the slide was placed in a preheated microscope chamber and maintained at 37 °C throughout the experiment.

***CLSM image deconvolution*** ***using a theoretical PSF:*** Confocal fluorescence microscopy images were deconvolved to improve spatial resolution and contrast by reducing the contribution of out-of-focus light. Deconvolution was performed using a theoretical point spread function (PSF) generated based on the optical parameters of the imaging system.

Theoretical PSFs were calculated using the PSF Generator Plugin in Fiji^3^ assuming diffraction-limited imaging conditions and using the system’s numerical aperture (NA), emission wavelength, refractive index of the immersion medium, and pixel sampling rate as inputs. For Cy5-labeled samples, the emission wavelength was set to 670 nm, corresponding to the fluorescence maximum of Cy5. The confocal system employed a 63×/1.40 NA oil immersion objective, and images were acquired with a pixel size of 9 nm, satisfying the Nyquist sampling criterion. The refractive index of the immersion oil and the sample mounting medium was taken as 1.518. The Born and Wolf 3D optical model was used with the accuracy of computation set to “Better”. For the deconvolution 2D images were used. Therefore, a 2D average of the 3D generated PSF was used as approximation.

Deconvolution was carried out using the DeconvolutionLab2 in Fiji^4^ with the Richardson-Lucy algorithm. A total of 10 iterations were applied. Regularization and background levels were optimized automatically using the built-in parameter estimation routines. The resulting deconvolved images exhibited enhanced signal-to-noise ratio and apparent resolution, enabling more reliable quantification of fibril thickness and morphology. Importantly, no nonlinear intensity rescaling or artificial sharpening filters were applied after deconvolution. All quantitative image analyses were performed on the deconvolved datasets to ensure consistency across samples.

### 1.1.7. Dynamic Light Scattering (DLS)

Dynamic light scattering (DLS) measurements of the various polymers (3mM **PEG-Tz1**, **PEG-Tz2**, **PEG-Nb** or PEG-OH) were performed using a Malvern Zetasizer Nano ZS ZEN3500. The laser wavelength was 633 nm and the scattering angle was 173^o^.

## **1.2 Synthetic Procedures**

The reported compounds used in this study were synthesized according to the published protocols listed below:

- *benzyl (8-aminooctyl)carbamate* ***(1)*** *and compound* ***SQ****^1^*
- *2-(4-(6-methyl-1,2,4,5-tetrazin-3-yl)phenyl)acetic acid* ***(Tz1)****^5^*
- *2-(4-(1,2,4,5-tetrazin-3-yl)phenyl)acetic acid* ***(Tz2)****^6^*
- *2,5-dioxopyrrolidin-1-yl 2-(4-(6-methyl-1,2,4,5-tetrazin-3-yl)phenyl)acetate* ***(Tz1-NHS)****^5^*
- *2,5-dioxocyclopentyl 2-(4-(1,2,4,5-tetrazin-3-yl)phenyl)acetate* ***(Tz2-NHS)****^6^*
- *Compound* ***SQ-Trt****^7^*

### 1.2.1 4-arm PEG Functionalization (PEG-Tz1, PEG-Tz2 and PEG-Nb)

***Synthesis of PEG-Nb using HCTU***

5-Norbornene-2-carboxylic acid (200 mg, 1.45 mmol, 18.0 equiv.) and HCTU (718 mg, 1.74 mmol, 21.6 equiv.) were dissolved in anhydrous DMF and stirred for 30 min. Thereafter, 4-arm PEG amine (804 mg, 0.08 mmol, 1.0 equiv.) and DIPEA (0.94 mL, 5.4 mmol, 60.0 equiv.) were added, and the reaction mixture was stirred for 2 days. The solvent was then evaporated at RT and the remaining product was redissolved in DCM (50 mL). The solution was washed 2 x with 1 M Na_2_H_2_PO_4_ (2 x 50 mL) and 2 x with brine (2 x 50 mL). The organic fraction was dried over Na_2_SO_4_, filtered, and concentrated *in vacuo* to obtain the crude solid. The reaction was dissolved in a minimum amount of DCM and reprecipitated 4x using diethyl ether (DCM : diethyl ether 1:10). Finally, the solid was dialyzed against H_2_O for 48 h and lyophilized to yield **PEG-Nb** as a white powder (790 mg, 0.08 mmol, 98%). ^1^H-NMR (500 MHz, CDCl_3_) δ 6.22 (dd, *J* = 5.7, 3.1 Hz, 0.5H), 6.13 (dd, *J* = 5.7, 3.0 Hz, 0.5H), 6.09 (dd, *J* = 5.6, 3.1 Hz, 0.5H), 5.97 (dd, *J* = 5.7, 2.8 Hz, 0.5H), 3.79-3.39 (m, 224H). The degree of functionalization was calculated as 95% based on ^1^H-NMR.

***General synthetic procedure of PEG-Tz1 and PEG-Tz2 via NHS activation***

**Tz-NHS** (16.0 equiv.), 4-arm PEG amine (1.0 equiv.) and DIPEA (5.0 equiv.) were dissolved in anhydrous DMF (2-4 mL) under an inert atmosphere. The reaction mixture was stirred overnight at RT. Thereafter, the solvent was evaporated at RT, and the remaining product was redissolved in a minimum amount of DCM. Precipitation from diethyl ether (DCM : diethyl ether 1:10) 4x afforded the product.

***Synthesis of PEG-Tz1 via NHS activation***

**Tz1-NHS** (77 mg, 0.23 mmol, 16.0 equiv.), 4-arm PEG amine (146 mg, 0.015 mmol, 1.0 equiv.) and DIPEA (12.7 μL, 0.07 mmol, 5.0 equiv.) were reacted in anhydrous DMF (4.0 mL) to yield **PEG-Tz1** as a pink powder (146 mg, 0.015 mmol, 99 %). ^1^H-NMR (400 MHz, CDCl_3_) δ 8.55 (d, *J* = 8.4 Hz, 2H), 7.53 (d, *J* = 8.4 Hz, 2H), 3.74 – 3.51 (m, 224H), 3.09 (s, 3H). The degree of functionalization was calculated as 94% based on ^1^H-NMR.

***Synthesis of PEG-Tz2 via NHS activation***

**Tz2-NHS** (52 mg, 0.17 mmol, 16.0 equiv.), 4-arm PEG amine (105 mg, 0.010 mmol, 1.0 equiv.) and DIPEA (9.0 μL, 0.04 mmol, 5.0 equiv.) were reacted in anhydrous DMF (2.0 mL) to yield **PEG-Tz2** as a red powder (104 mg, 0.010 mmol, 99 %). ^1^H-NMR (400 MHz, CDCl_3_) δ 10.22 (s, 1H), 8.59 (d, *J* = 8.4 Hz, 2H), 7.56 (d, *J* = 8.3 Hz, 2H), 3.82-3.40 (m, 224H). The degree of functionalization was calculated as 87% based on ^1^H-NMR.

***2-(2-(2-(2-hydroxyethoxy)ethoxy)ethoxy)ethyl 4-methylbenzenesulfonate (2)***

Tetraethylene glycol (81.50 g, 419.61 mmol, 8.0 equiv.) was transferred to a round-bottom flask and dissolved in THF (20 mL). The solution was stirred in an ice bath and treated with 20 mL of aqueous sodium hydroxide (3.14 g, 78.67 mmol, 1.5 equiv.). Then, a solution of p-toluene sulfonyl chloride (Ts-Cl) (10.0 g, 52.45 mmol, 1.0 equiv.) in THF (60 mL) was added dropwise over two hours to the reaction mixture in an ice bath. The reaction was stirred overnight at room temperature. TLC confirmed the reaction completion, and then THF was removed using a rotary evaporator. The crude reaction mixture was dissolved in 200 mL of DCM and washed with water (3 x 100 mL). The compound was then extracted with DCM. The organic layer was dried over Na_2_SO_4_, filtered, and the solvent was removed *in vacuo*. The crude product was further purified by silica column chromatography using 5% MeOH : DCM. The fractions were concentrated by rotary evaporation to obtain an oil-like product **2** (16.2g, 46.5 mmol, 89%). ^1^H NMR (400 MHz, CDCl_3_) δ 7.81 (d, J = 8.3 Hz, 2H), 7.35 (d, J = 8.0 Hz, 2H), 4.24 – 4.11 (m, 2H), 3.77 – 3.54 (m, 15H), 2.45 (s, 3H). ^13^C NMR (75 MHz, CDCl_3_) δ 144.88, 133.01, 129.90, 128.04, 72.54, 70.70, 70.65, 70.50, 70.35, 69.31, 68.76, 61.78, 21.71.

***2-(2-(2-(2-azidoethoxy)ethoxy)ethoxy)ethan-1-ol (3)***

Sodium azide (6.53 g, 100.57 mmol. 5.0 equiv.) was added to a stirred solution of compound 2 (7.0 g, 20.11 mmol, 1.0 equiv.) in ethanol (80 mL). The reaction mixture was refluxed at 70 °C overnight. The reaction completion was confirmed by TLC. The resulting white solid was filtered off, and the ethanol was removed under reduced pressure. The crude residue was treated with water (100 mL) and extracted with dichloromethane (3 x 100 mL). The combined organic layers were dried over anhydrous Na_2_SO_4_, filtered, and concentrated *in vacuo*. The resulting oil-like product, **3** (4.2g, 19.1 mmol, 95%) was confirmed by NMR and used without further purification. ^1^H NMR (300 MHz, CDCl_3_) δ 3.77 – 3.65 (m, 12H), 3.61 (s, 2H), 3.41 (d, J = 10.1 Hz, 2H). ^13^C NMR (75 MHz, CDCl_3_) δ 72.55, 70.75, 70.72, 70.64, 70.39, 70.10, 61.81, 50.72.

### 1.2.2. Synthetic route of the sulfo-Cyanine5 dye Functionalized SQ Monomer (SQ-Cy5)

**Scheme S2.** Synthesis route of SQ-Cy5.

***2-(2-(2-(2-aminoethoxy)ethoxy)ethoxy)ethan-1-ol (4)***

Triphenylphosphine (PPh_3_) (7.58 g, 28.90 mmol, 1.5 equiv.) was added to a stirred solution of compound 3 (4.22 g, 19.26 mmol, 1.0 equiv.) in THF (80 mL). The reaction mixture was stirred at room temperature overnight. The reaction completion was confirmed by TLC. THF was removed *in vacuo*. The crude residue was treated with water (100 mL) and extracted with water and washed with toluene (3 x 100 mL). The combined aqueous layers were concentrated *in vacuo*. The resulting oil-like product, **4** (3.3g, 17.3 mmol, 90%) was confirmed by NMR and used without further purification. ^1^H NMR (300 MHz, CDCl_3_) δ 3.74 – 3.69 (m, 2H), 3.65 (d, J = 4.3 Hz, 8H), 3.61 – 3.51 (m, 4H). ^13^C NMR (75 MHz, CDCl_3_) δ 73.04, 72.63, 70.57, 70.48, 70.17, 70.05, 61.35, 41.24.

***tert-butyl (2-(2-(2-(2-hydroxyethoxy)ethoxy)ethoxy)ethyl)carbamate (5)***

Triethylamine (Et_3_N) (2.4g, 23.72 mmol, 1.5 equiv.) was added to a stirred solution of compound 4 (3.0 g, 15.81 mmol, 1.0 equiv.) in DCM (30 mL). The reaction mixture was stirred in an ice-bath and allowed to reach at 4°C. Afterwards, di-tert-butyl dicarbonate (Boc_2_O) (4.14 g, 18.98 mmol, 1.2 equiv.) was added. The reaction mixture was allowed to reach room temperature and stirred overnight. Completion of the reaction was confirmed by TLC, prior to removal of DCM *in vacuo*. The crude product was further purified by silica column chromatography using 5% MeOH : DCM. The fractions were concentrated by rotary evaporation to obtain an oil-like product **5**. (3.3 g, 11.5 mmol, 73%) ^1^H NMR (300 MHz, CDCl_3_) δ 3.76 – 3.68 (m, 4H), 3.68 – 3.58 (m, 8H), 3.53 (dd, J = 5.5, 4.5 Hz, 2H), 3.31 (dd, J = 5.5, 4.5 Hz, 2H), 1.44 (s, 9H). ^13^C NMR (75 MHz, CDCl_3_) δ 156.26, 79.12, 70.66, 70.49, 70.30, 70.13, 69.62, 69.44, 69.26, 69.09, 40.60, 28.49, 27.44.

***benzyl (2,2-dimethyl-4-oxo-3,8,11,14-tetraoxa-5-azahexadecan-16-yl) octane-1,8-diyldicarbamate (6)***

Compound 5 (3.0 g, 10.85 mmol, 1.0 equiv.) was dissolved in CHCl_3_ (30 mL) and then activated with CDI (2.01 g, 12.42 mmol, 1.1 equiv.) for two hours at room temperature. TLC was used to confirm the activated product, followed by the addition of compound 1 (3.18 g, 11.39 mmol, 1.05 equiv.) and DIPEA (2.0 g, 15.53 mmol, 1.4 equiv.) to the reaction mixture prior to refluxing overnight. Completion of the reaction was confirmed by TLC, prior to removal of the solvent *in vacuo*. The crude product was further purified by silica column chromatography using 5% MeOH : EtOAc and the fractions were concentrated by rotary evaporation to obtain an oil-like product **6** (3.0g, 5.21 mmol, 48%). ^1^H NMR (300 MHz, CDCl_3_) δ 7.36 (d, J = 5.0 Hz, 5H), 5.10 (s, 3H), 4.22 (t, J = 4.7 Hz, 2H), 3.66 (s, 11H), 3.54 (t, J = 5.1 Hz, 2H), 3.31 (d, J = 15.5 Hz, 2H), 3.24 – 3.07 (m, 4H), 1.44 (s, 13H), 1.29 (s, 9H). ^13^C NMR (75 MHz, CDCl_3_) δ 156.46, 156.09, 136.71, 128.57, 128.19, 128.14, 79.24, 70.64, 70.61, 70.56, 70.31, 69.77, 66.62, 63.84, 41.11, 41.03, 40.42, 29.96, 29.17, 28.48, 26.67.

***2,2-dimethyl-4-oxo-3,8,11,14-tetraoxa-5-azahexadecan-16-yl (8-((2-butoxy-3,4-dioxocyclobut-1-en-1-yl)amino)octyl)carbamate (8)***

Compound 6 (0.5 g, 0.8375 mmol, 1.0 equiv.) was dissolved in dry CHCl_3_ (5 mL) and dry MeOH (5 mL), and 10 mol% Pd/C (50 mg) was suspended in the reaction mixture. The reaction mixture was stirred under inert N_2_ atmosphere, and triethyl silane (HSiEt_3_) (1.1 g, 10.05 mmol, 12.0 equiv.) was added dropwise before stirring an additional 2 hours at room temperature. The progress of the reaction was monitored by TLC. After the reaction was completed, the crude was filtered through a celite bed and washed with methanol (2 x 10 mL). The solvent was then removed *in vacuo*. The oil-like product **7** was used was subsequently dissolved in CHCl_3_ (5 mL), and DIPEA (0.28g, 2.164 mmol, 2.5 mmol) was added. Dibutyl squarate (0.269 g, 1.19 mmol, 1.4 equiv.) was added to the reaction mixture above. The reaction was stirred at room temperature for 4 hours. The reaction completion was confirmed by TLC. The solvent was removed from the reaction mixture, and the crude product was further purified by silica column chromatography using 1-5% Pet-ether: EtOAc gradient elution. The fractions were concentrated by rotary evaporation to obtain an oil-like product **8** (0.46 g, 0.75 mmol, 75%) ^1^H NMR (300 MHz, CDCl_3_) δ 5.10 (s, 1H), 4.92 (s, 1H), 4.74 (t, J = 6.3 Hz, 2H), 4.21 (t, J = 4.7 Hz, 2H), 3.64 (d, J = 9.0 Hz, 11H), 3.54 (t, J = 5.1 Hz, 2H), 3.42 (d, J = 6.8 Hz, 1H), 3.31 (q, J = 5.5 Hz, 2H), 3.15 (q, J = 6.7 Hz, 2H), 1.96 (s, 1H), 1.77 (q, J = 6.8, 6.4 Hz, 2H), 1.59 (d, J = 7.0 Hz, 2H), 1.44 (s, 13H), 1.30 (d, J = 5.1 Hz, 10H), 0.97 (t, J = 7.4 Hz, 3H). ^13^C NMR (75 MHz, CDCl3) δ 189.59, 183.08, 177.57, 172.55, 156.57, 156.19, 79.33, 73.53, 70.69, 70.66, 70.61, 70.38, 69.81, 63.93, 44.95, 41.04, 40.49, 32.13, 30.70, 29.98, 29.82, 29.25, 29.11, 28.55, 26.75, 26.66, 26.36, 18.76, 14.79, 13.80.

***Compound SQ-NHBoc***

**SQ-Trt** (0.2 g, 0.154 mmol, 1.0 equiv.) was dissolved in 50 TFA : 50 DCM (5 mL). The reaction mixture was stirred for 30 minutes at RT and the reaction completion was confirmed by TLC. The excess TFA was co-evaporated by sequential addition of DCM under N_2_ flow. Afterwards, the crude product was dissolved in CHCl_3_ (10 mL), followed by the addition of compound 8 (0.123 g, 0.2 mmol, 1.3 equiv.) and DIPEA (0.1 mL, 0.574 mmol, 3.6 equiv.). The reaction mixture was then stirred and refluxed overnight. After the reaction was completed, the solvent was then removed *in vacuo*. The oil-like product **SQ-NHBoc** was used for the next reaction without further purification. LC-MS (ESI) m/z: [M+H]^+^ calcd. for C_76_H_133_N_11_O_25_ 1600.95; found 1600.53

***Compound SQ-Cy5***

**SQ-NHBoc** (5.1 mg, 3.22 mmol, 1.0 equiv.) was dissolved 50 TFA : 50 DCM (1 mL). The mixture was stirred for 30 minutes at RT and the reaction completion was confirmed by TLC. The excess TFA was co-evaporated with sequential addition of DCM under N_2_ flow. Afterwards, the crude product was dissolved in DMSO (5 mL), followed by the addition of Sulfo-Cy5-NHS (2.8 mg, 3.54 mmol, 1.1 equiv.) and TEA (0.33 ml, 2.4 mmol, 0.75 equiv.). The reaction mixture was then stirred at RT overnight. After the reaction was completed, the solvent was then removed under N_2_ flow. The product was purified by HPLC using mass detection and lyophilized overnight to obtain compound **SQ-Cy5** as purple solid. LC-MS (ESI) m/z: [M+H]^+^ calcd. for C_103_H_160_N_13_O_30_S_2_^-^ 2124.08; found 1063.47 ([M+H]^+^)/2.

### 1.2.3. ^1^H-NMR and ^13^C-NMR Spectra


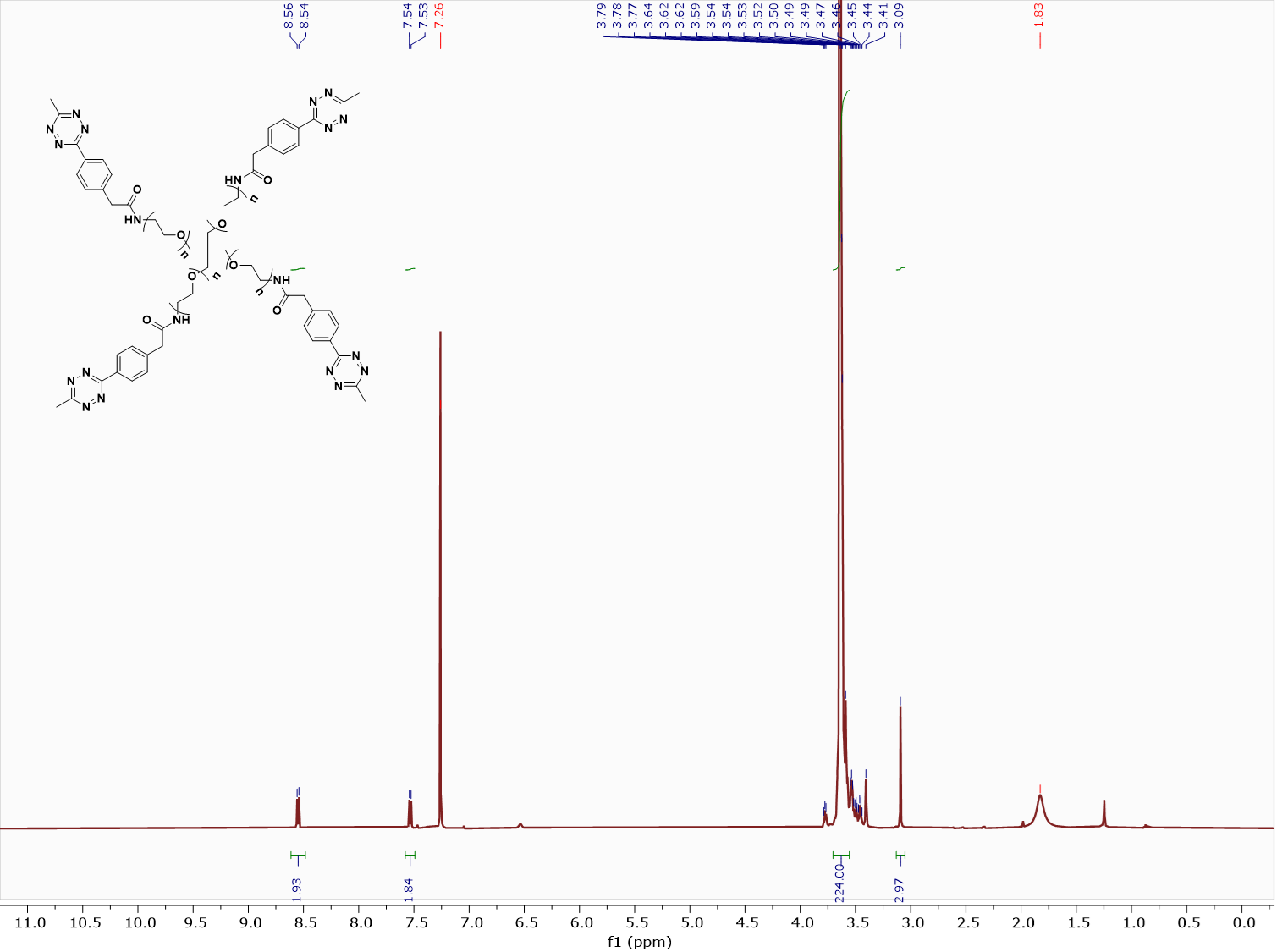


**Figure S.A1**. ^1^H-NMR (500 MHz, 298K, CDCl_3_) spectrum of **PEG-Tz1**.

***
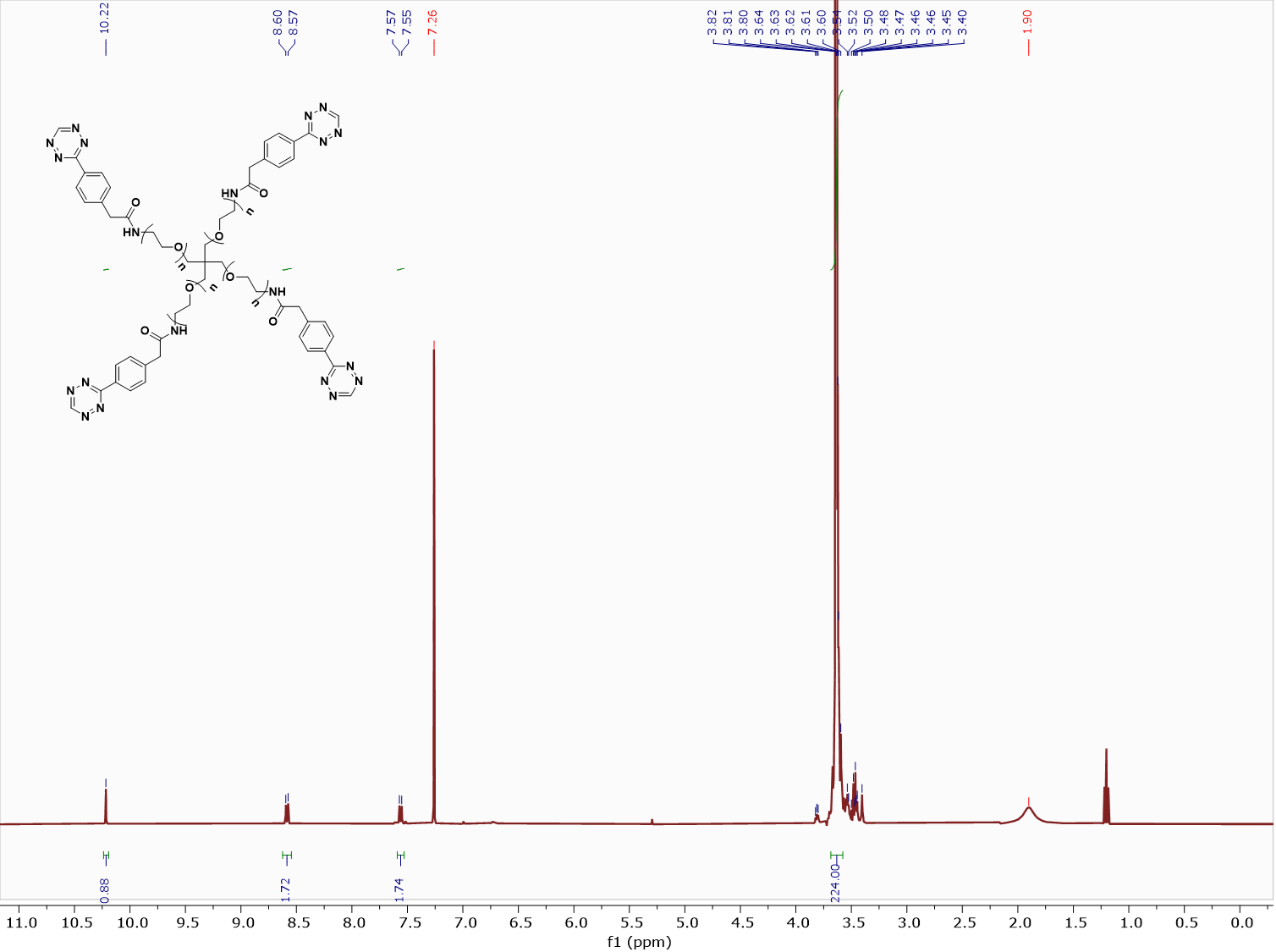
***

**Figure S.A2**. ^1^H-NMR (400 MHz, 298K, CDCl_3_) spectrum of **PEG-Tz2**.

*
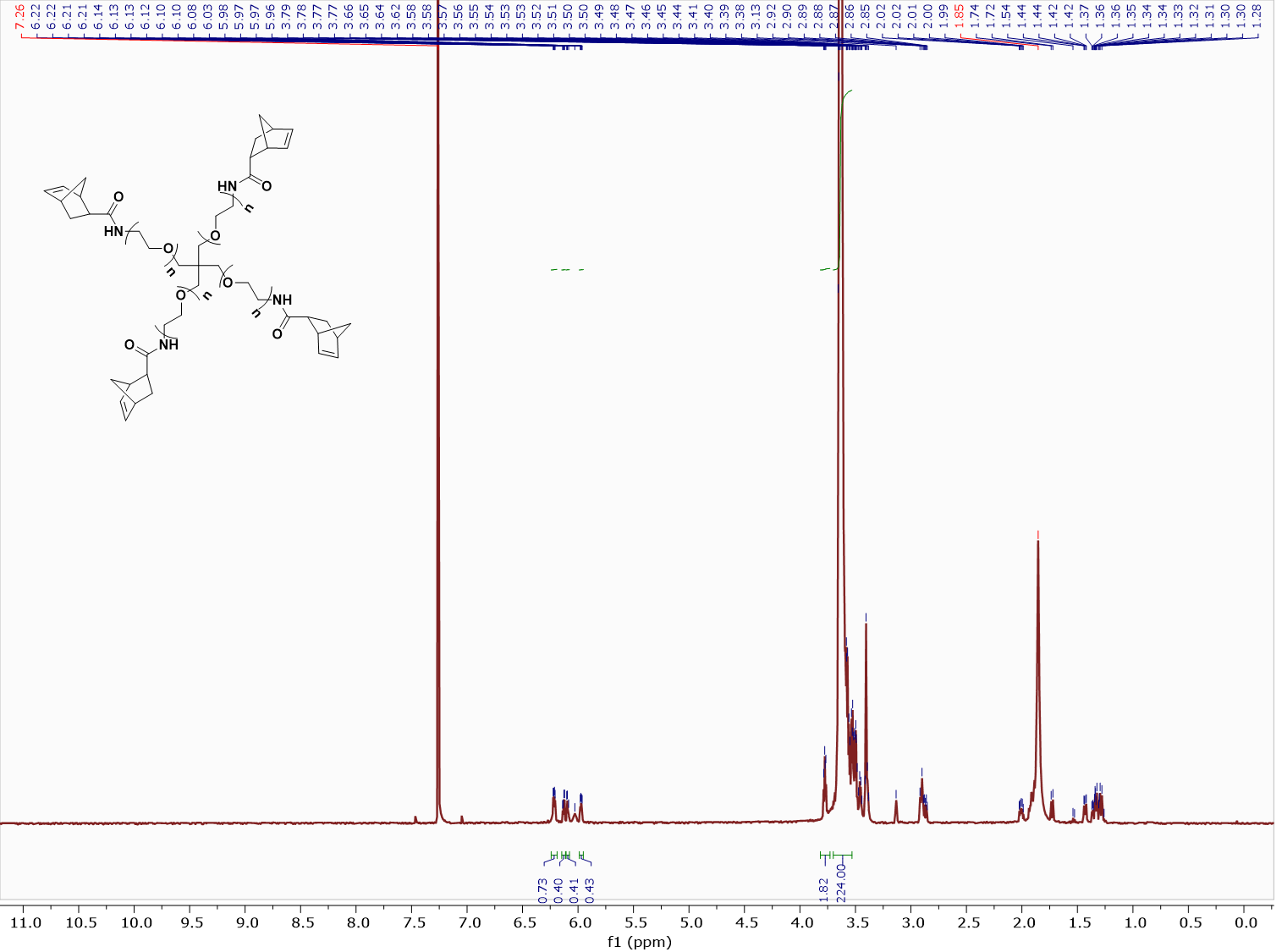
*

**Figure S.A3.** ^1^H-NMR (500 MHz, 298K, CDCl_3_) spectrum of **PEG-Nb**.


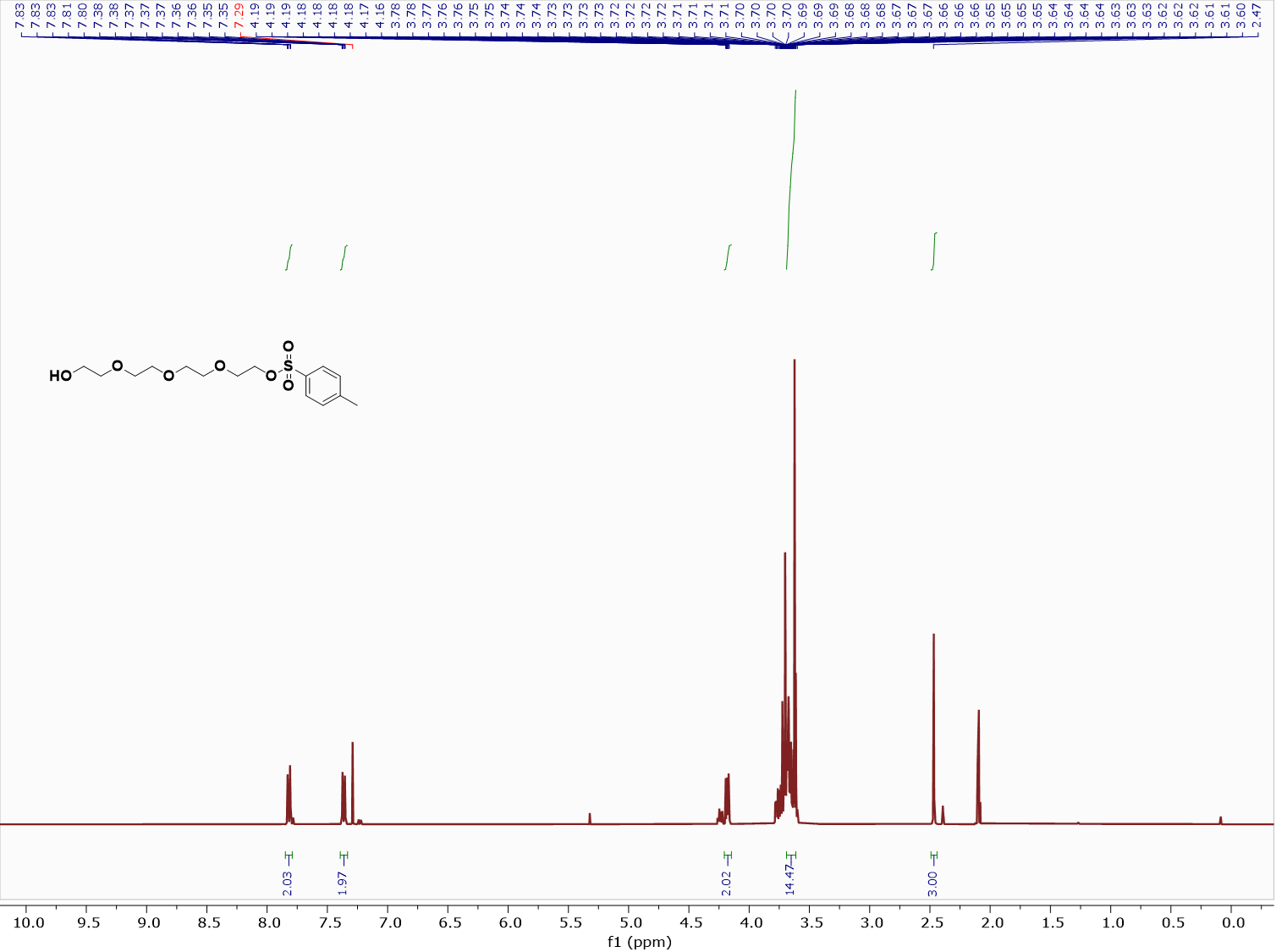


**Figure S.A4.** ^1^H-NMR (400 MHz, 298K, CDCl_3_) spectrum of **2**.

**Figure S.A5**. ^13^C-NMR (100 MHz, 298K, CDCl_3_) spectrum of **2**.


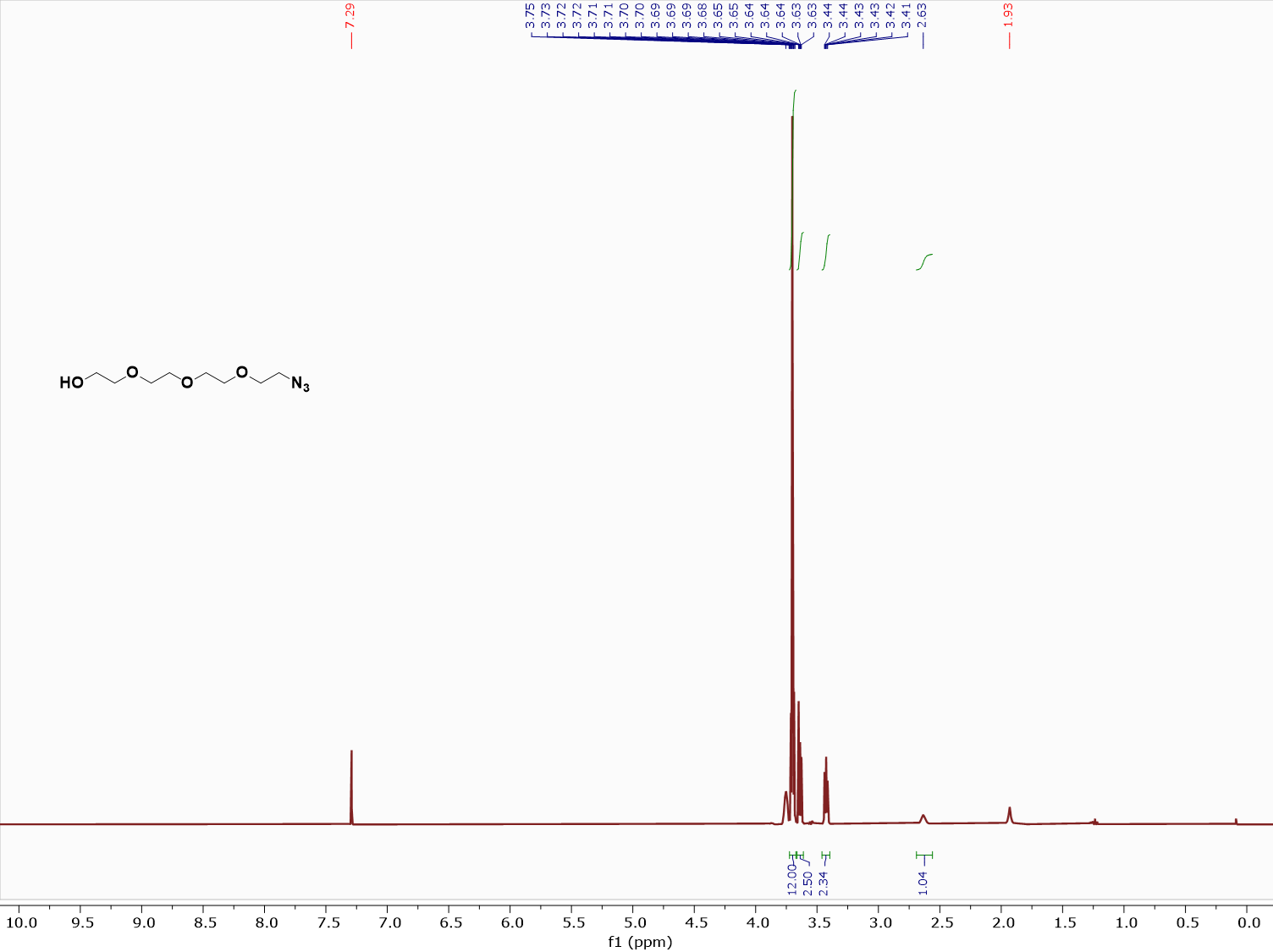


**Figure S.A6**. ^1^H-NMR (400 MHz, 298K, CDCl_3_) spectrum of **3**.


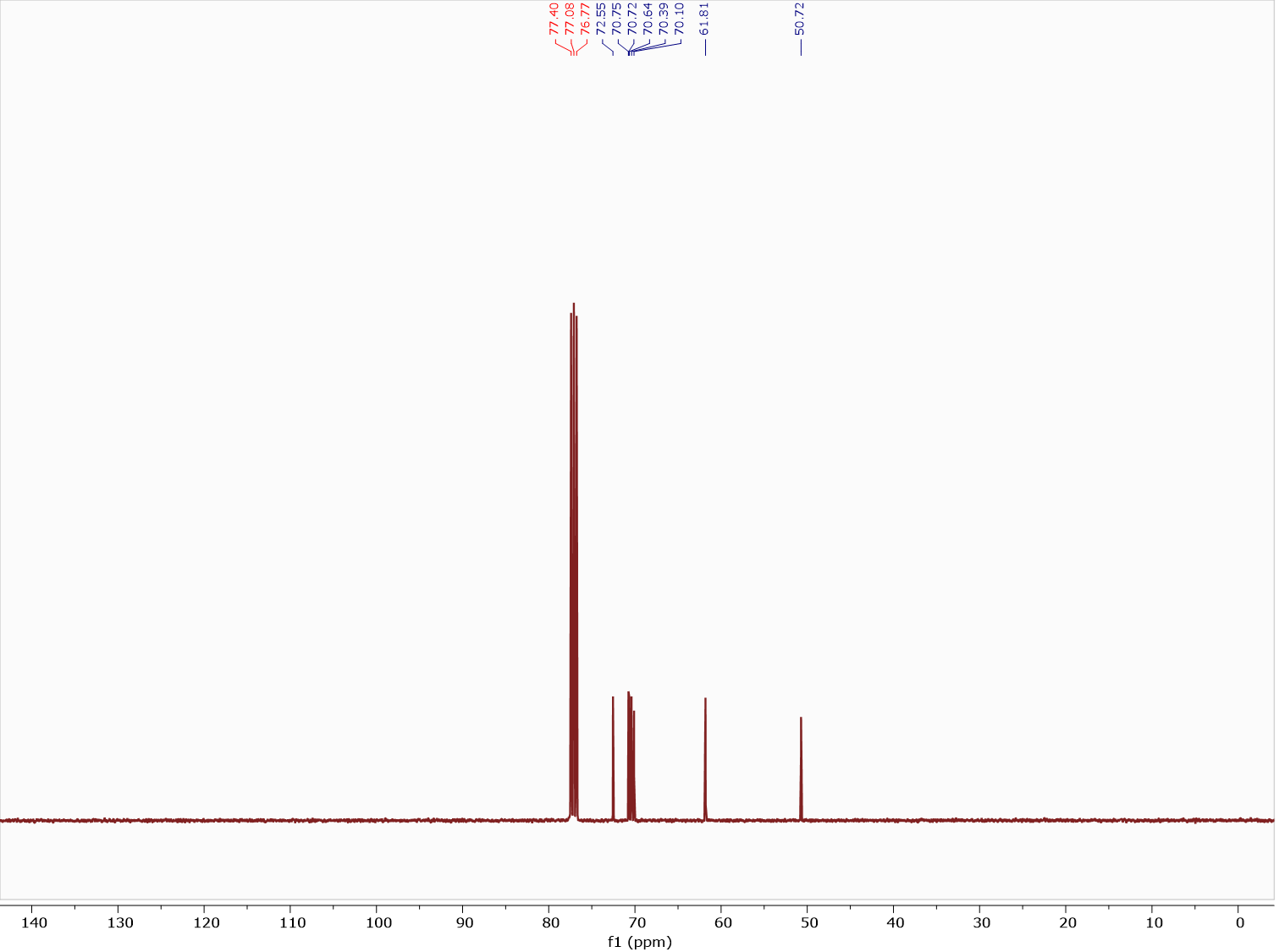


**Figure S.A7.** ^13^C-NMR (100 MHz, 298K, CDCl_3_) spectrum of **3**.


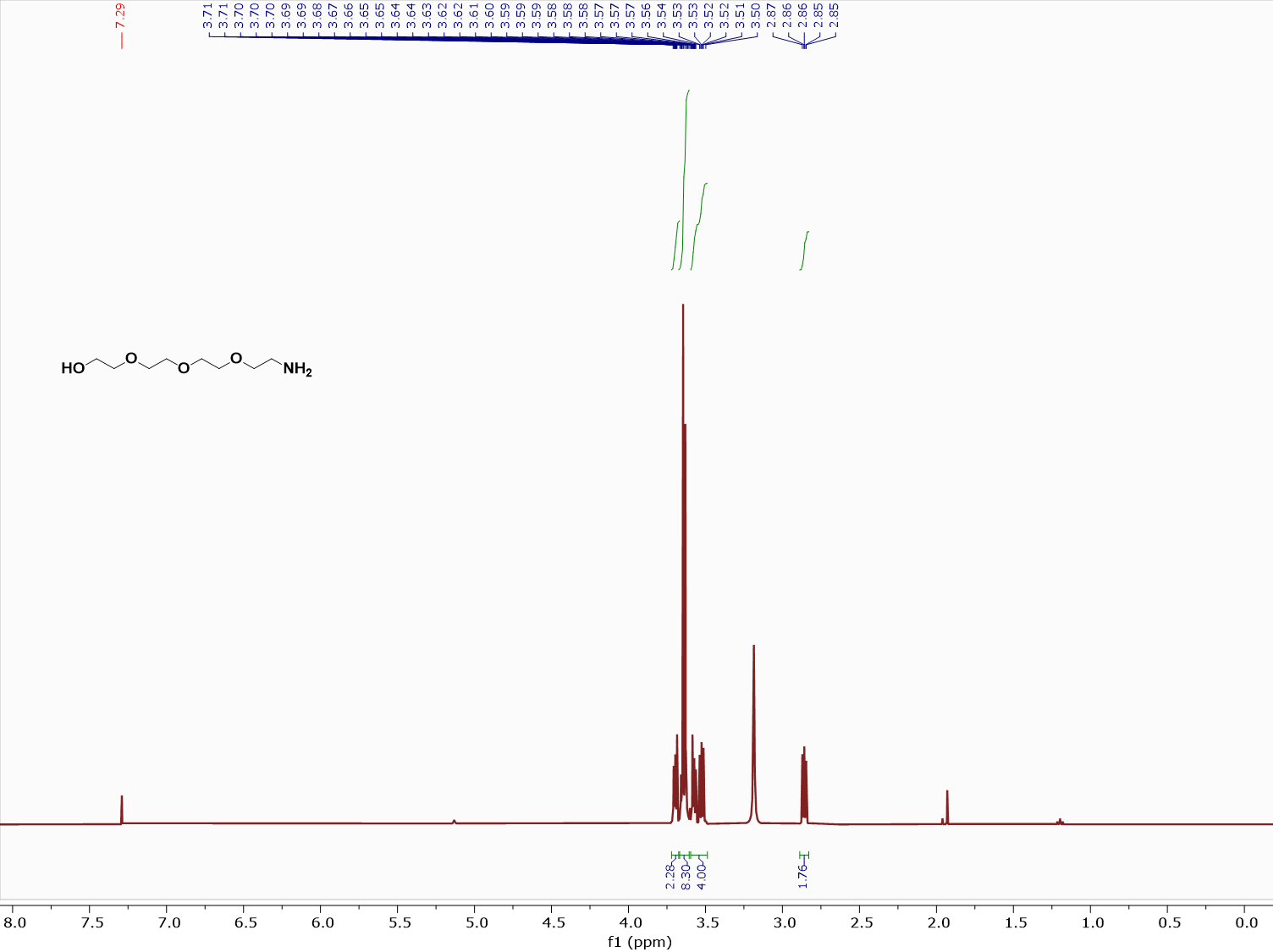


**Figure S.A8.** ^1^H-NMR (400 MHz, 298K, CDCl_3_) spectrum of **4**.

**Figure S.A9.** ^13^C-NMR (100 MHz, 298K, CDCl_3_) spectrum of **4**.


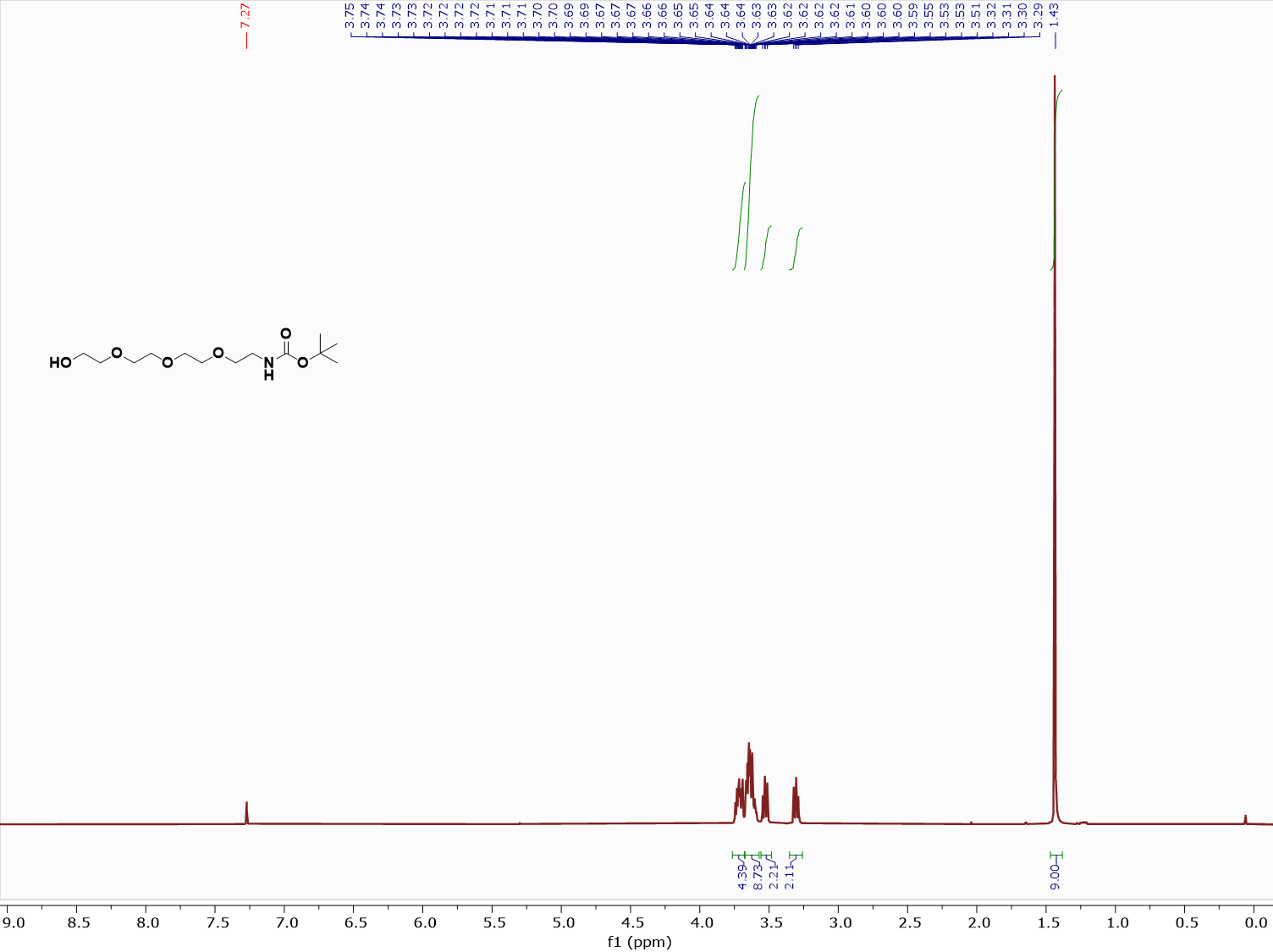


**Figure S.A10.** ^1^H-NMR (400 MHz, 298K, CDCl_3_) spectrum of **5**.

**Figure S.A11.** ^13^C-NMR (100 MHz, 298K, CDCl_3_) spectrum of **5**.


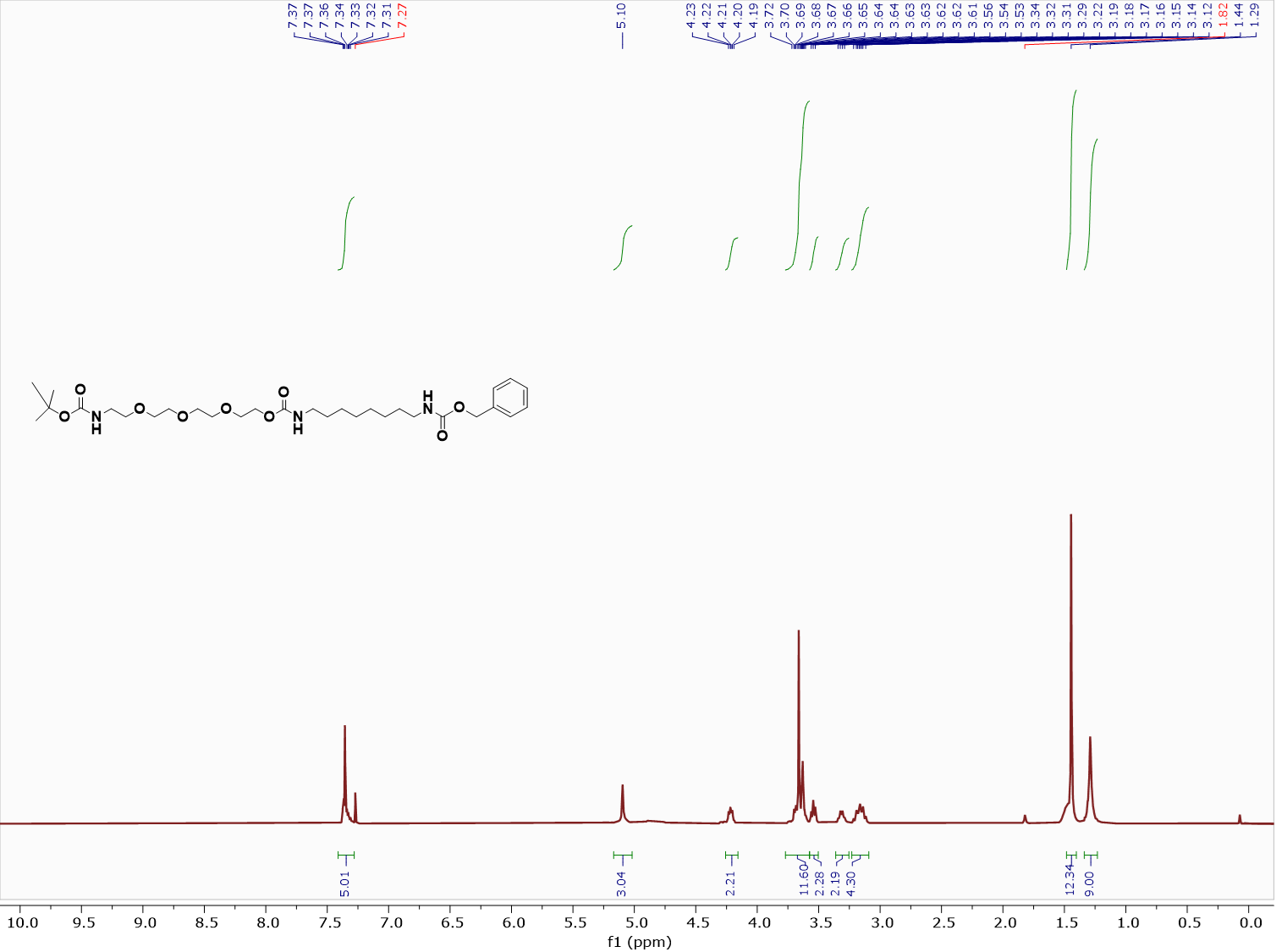


**Figure S.A12.** ^1^H-NMR (400 MHz, 298K, CDCl_3_) spectrum of **6**.


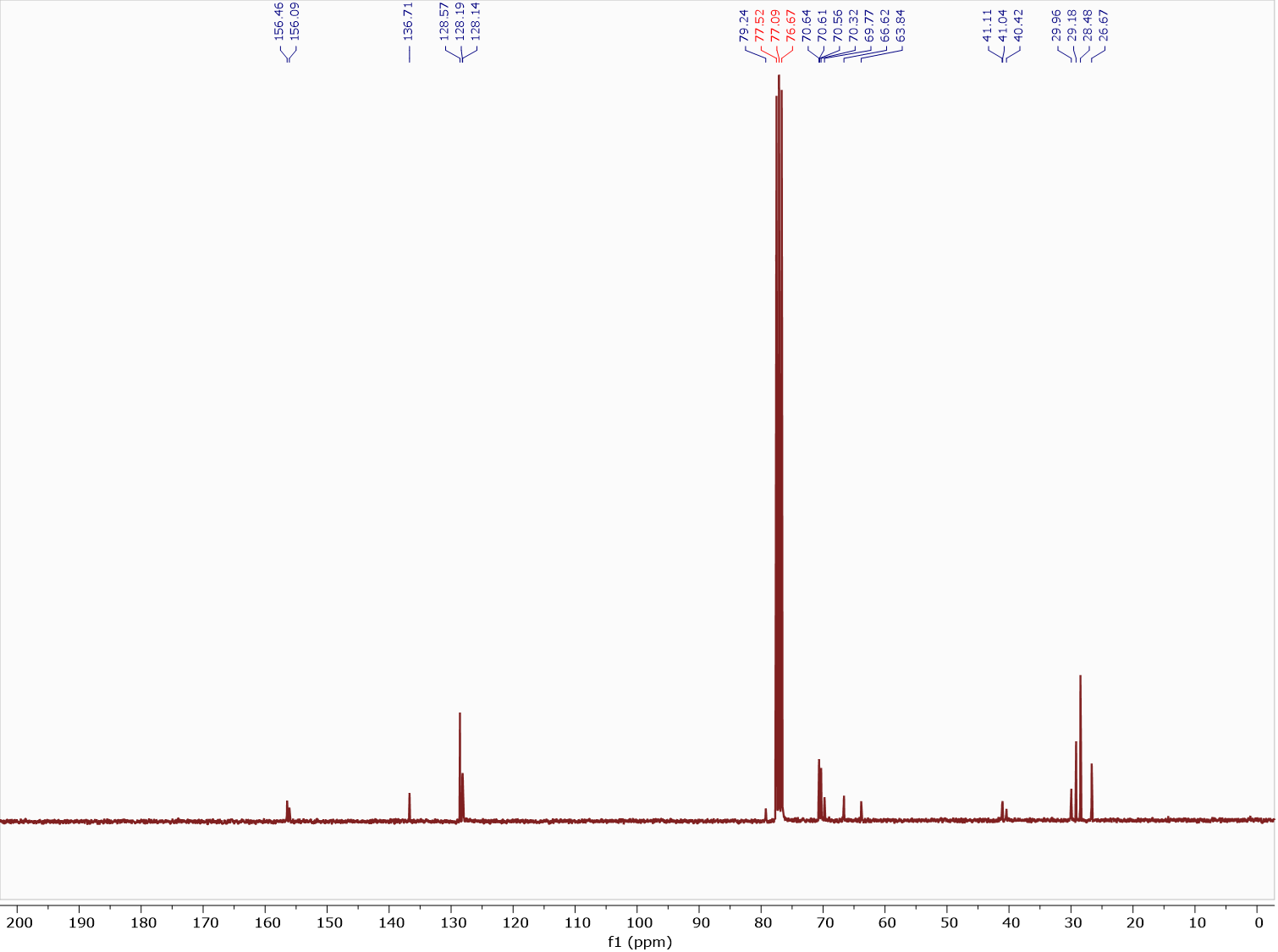


**Figure S.A13**. ^13^C-NMR (100 MHz, 298K, CDCl_3_) spectrum of **6**.


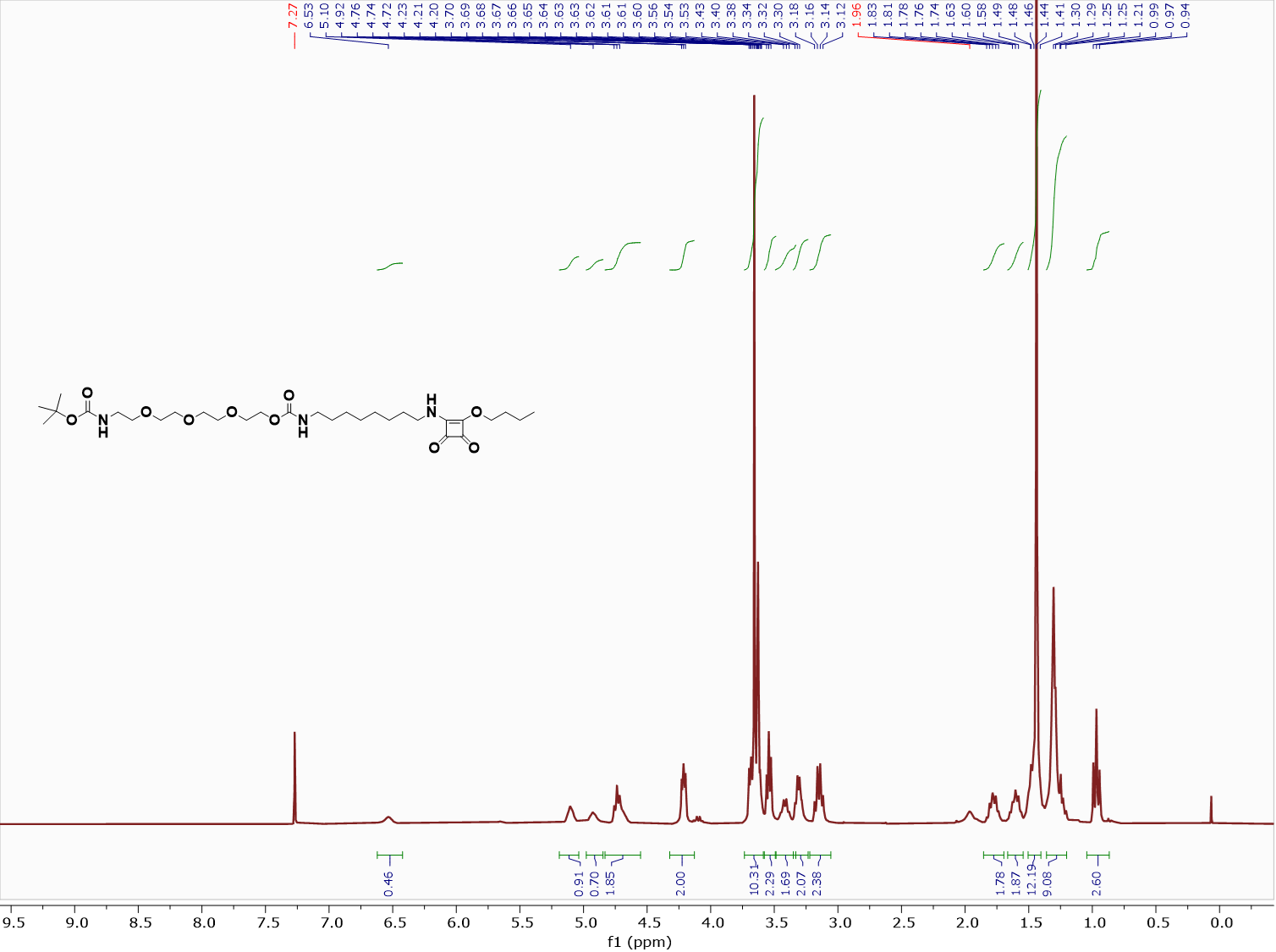


**Figure S.A.14.** ^1^H-NMR (400 MHz, 298K, CDCl_3_) spectrum of **8**.


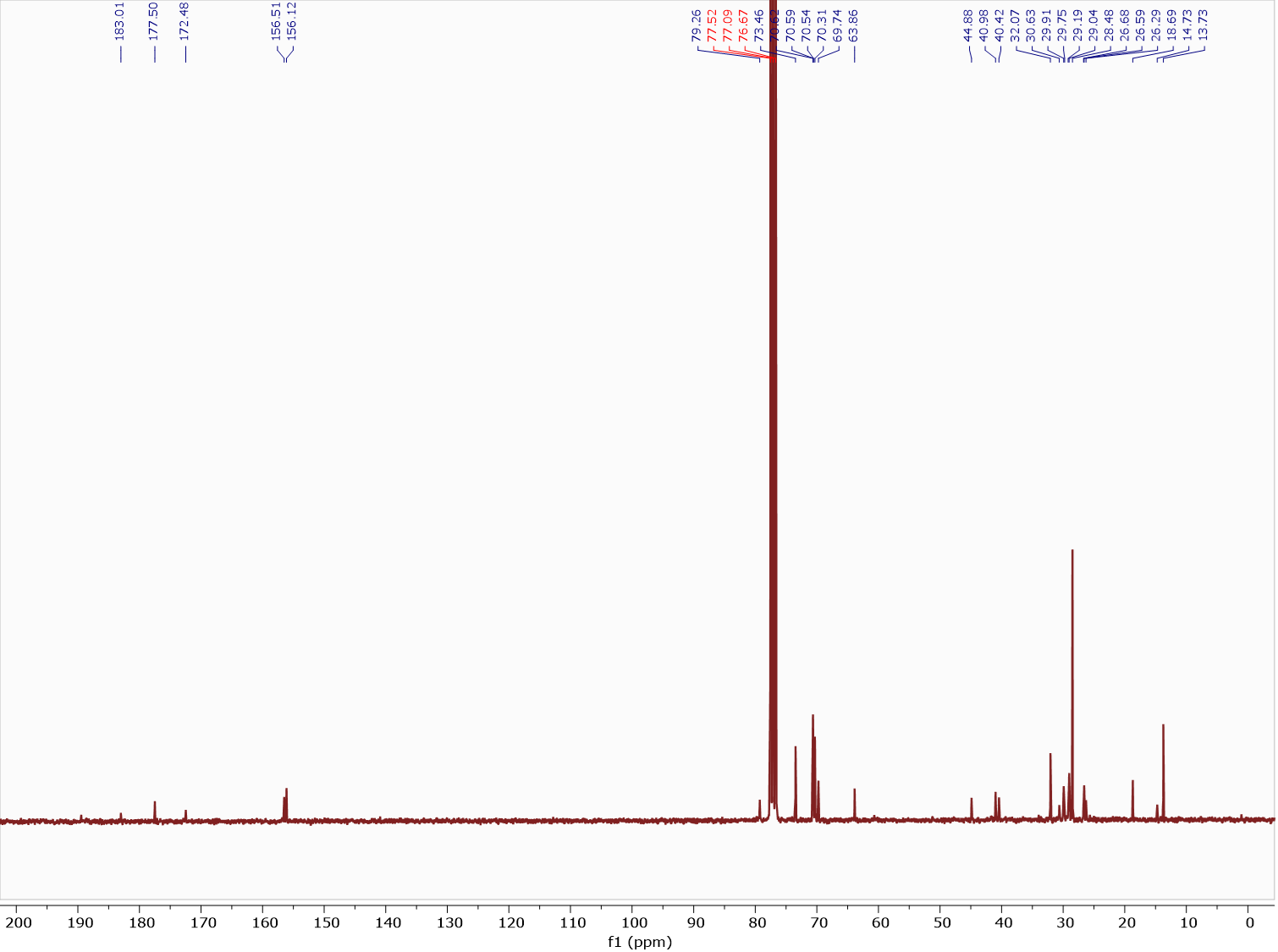


**Figure S.A15.** ^13^C-NMR (100 MHz, 298K, CDCl_3_) spectrum of **8**.

### 1.2.4. LC-MS Analysis


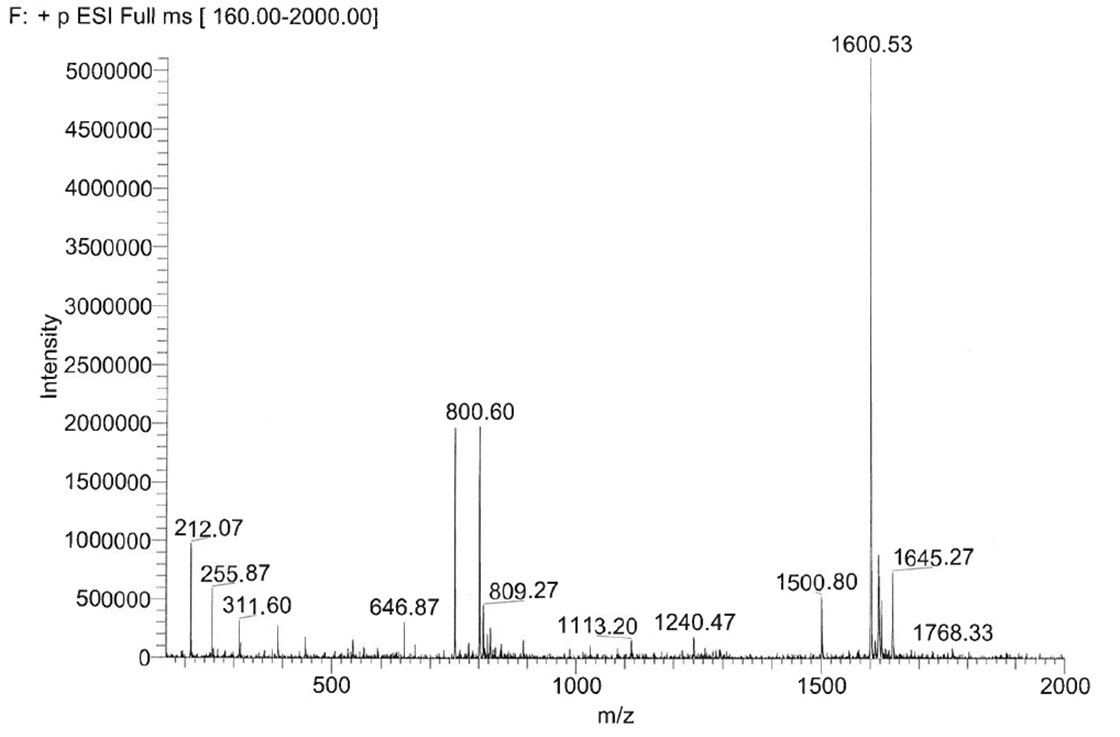


**Figure S.A16**. ESI Mass spectrum of compound **SQ-NHBoc** from LC-MS analysis.


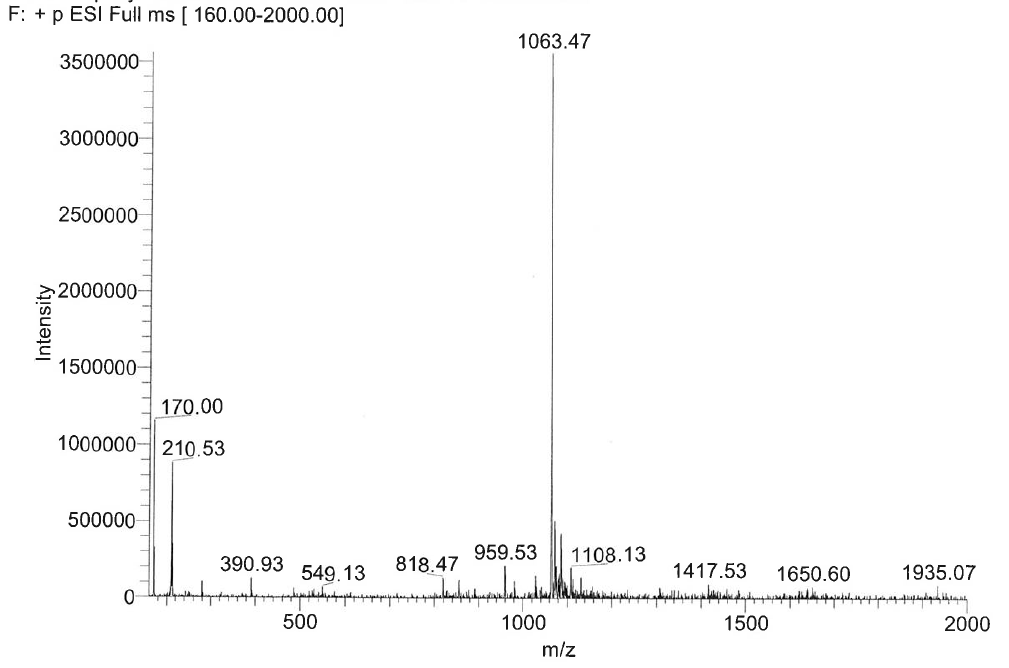


**Figure S.A17**. ESI Mass spectrum of compound **SQ-Cy5** from LC-MS analysis.

# 2. Additional Figures and Tables


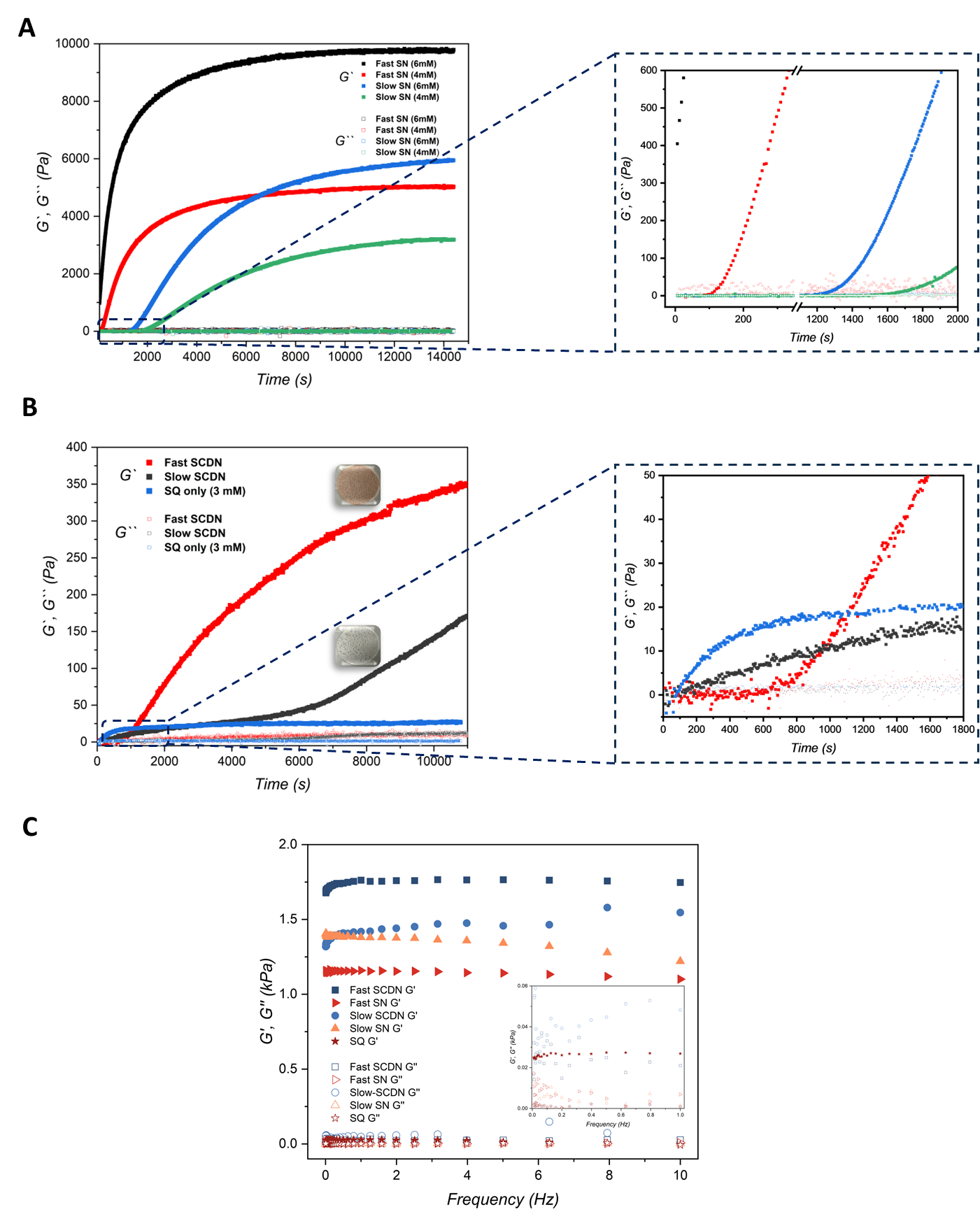


**Figure S1.** Time sweep measurements of SN (A) and SCDN (B) hydrogels. Oscillatory rheology measurements performed using 0.05% strain at 1 Hz and 37°C. SN: 4-6 mM **PEG-Tz1/Nb** or **PEG-Tz/ Nb**. SCDN: 3 mM **SQ** and 2 mM **PEG-Tz1/Nb** or **PEG-Tz2/Nb**. Frequency sweep measurements of SCDN and SN hydrogels (C) from 0.01-10 Hz with constant strain of 0.05%. SCDN: 3 mM **SQ** and 3 mM **PEG Tz1/Nb** or **PEG-Tz2/Nb**, SN: 3 mM **PEG-Tz1/Nb** or **PEG-Tz2/Nb**, **SQ**: 3 mM.

**
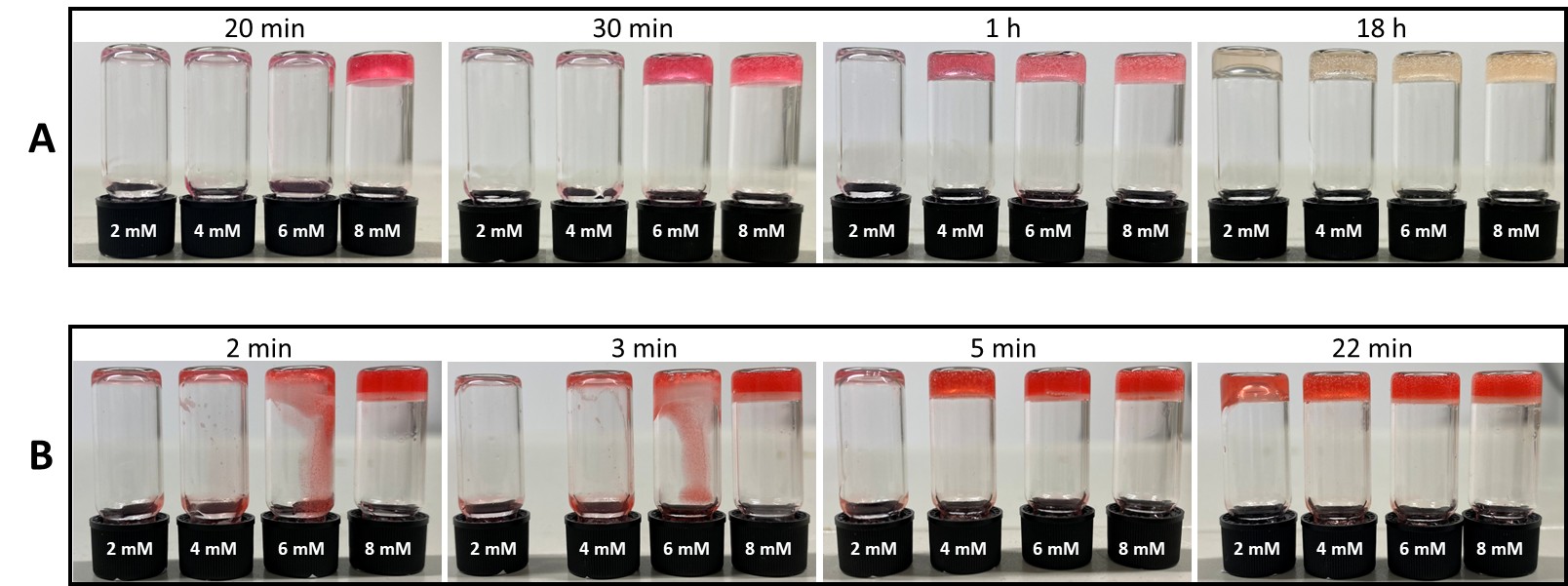
**

**Figure S2.** Gel inversion test of slow **PEG-Tz1/Nb** (A) and fast **PEG-Tz2/Nb** (B) forming hydrogels at different total polymer concentrations (2-8 mM). Incubation of the hydrogels were performed at 37°C in PBS.


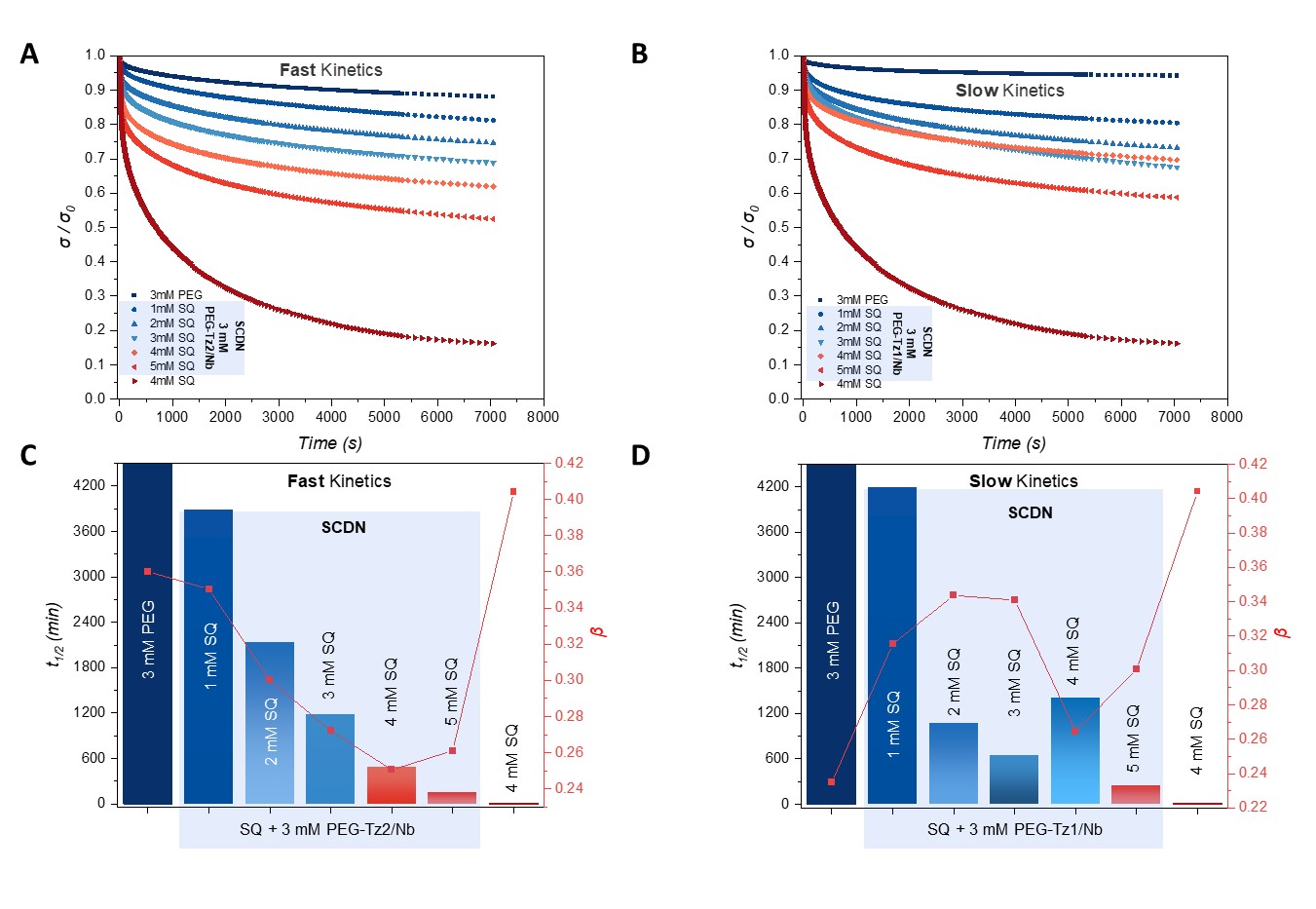


**Figure S3.** Stress-relaxation behavior of SCDNs with varying **SQ** concentration (1 - 5 mM) in the presence of fast and slow covalent network formation. Normalized stress-relaxation curves at 10% strain and 37 °C for hydrogels formed via fast (A) or slow (B) IEDDA crosslinking. Stress-relaxation half-time (t_1/2_, bars) and β parameter (red line, right axes) calculated from stretched exponential fits using Kohlrausch-Williams-Watts (KWW) equation, commonly used to quantify relaxation rates in hydrogel materials (C and D).


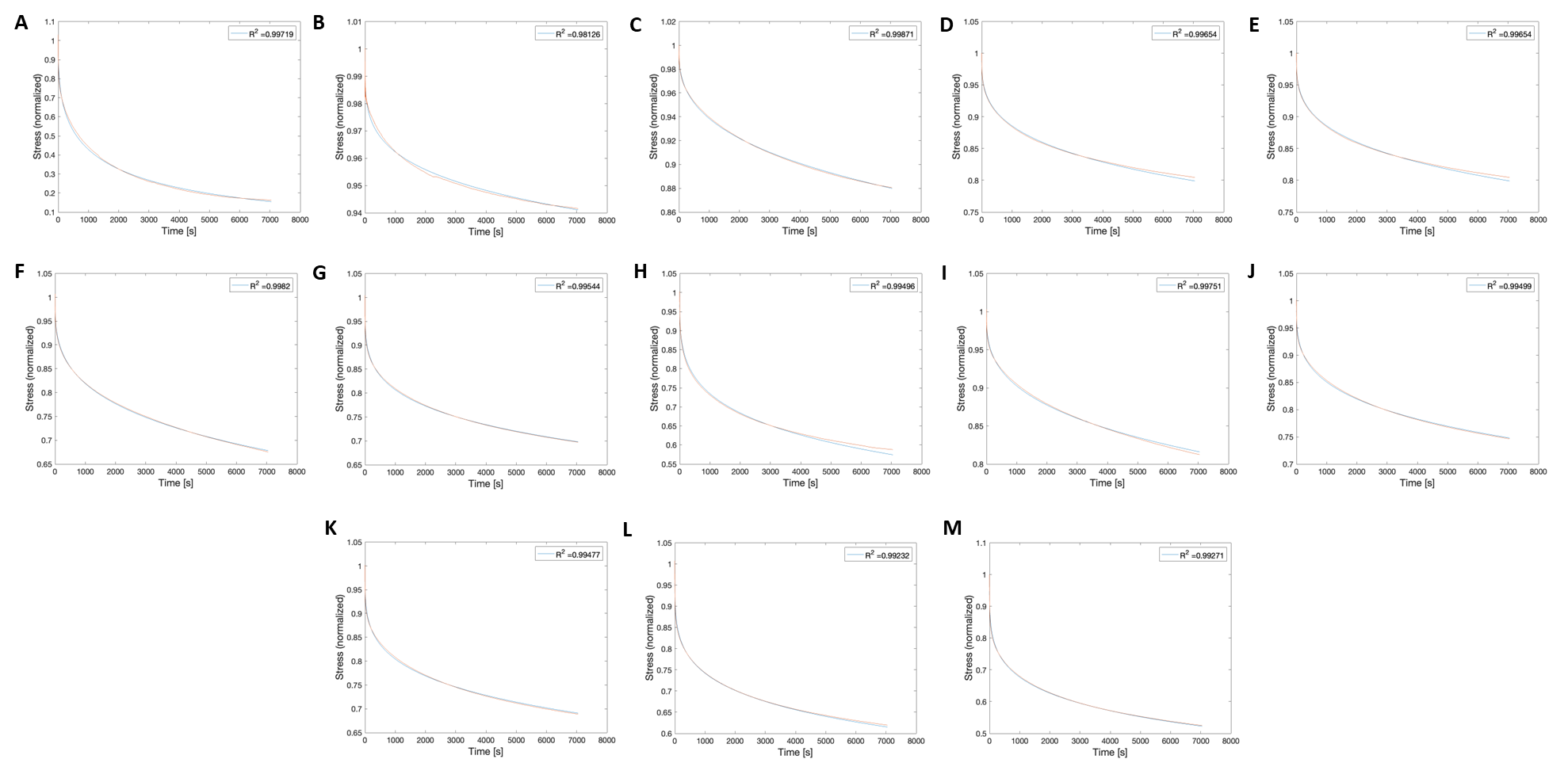


**Figure S4.** Results of the stress-relaxation curves fitted with stretched exponential KWW equation (equation S1) for the following hydrogels: A) 4 mM **SQ**, B) 3 mM **PEG-Tz1/Nb**, C) 3 mM **PEG-Tz2/Nb**, D) 1 mM **SQ** and 3 mM **PEG-Tz1/Nb**, E) 2 mM **SQ** and 3 mM **PEG-Tz1/Nb**, F) 3 mM **SQ** and 3 mM **PEG-Tz1/Nb**, G) 4 mM **SQ** and 3 mM **PEG-Tz1/Nb**, H) 5 mM **SQ** and 3 mM **PEG-Tz1/Nb**, I) 1 mM **SQ** and 3 mM **PEG-Tz2/Nb**, J) 2 mM **SQ** and 3 mM **PEG-Tz2/Nb**, K) 3 mM **SQ** and 3 mM **PEG-Tz2/Nb**, L) 4 mM **SQ** and 3 mM **PEG-Tz2/Nb**, and M) 5 mM **SQ** and 3 mM **PEG-Tz2/Nb**. The fitted parameters have been calculated using MathWorks-MATLAB and listed in Table S1.

**Table S1.** Fitted parameters of stress relaxation data for the various hydrogel compositions.

| **Sample** | **t_1/2_ (s)** | **β** | **τ_k_** | **R^2^ value** |
| --- | --- | --- | --- | --- |
| **4 mM SQ** | 608.3 | 0.4042 | 1506 | 0.99719 |
| **3 mM PEG-Tz1/Nb** | 2.24x10^8^ | 0.2349 | 1.07x10^9^ | 0.98126 |
| **3 mM PEG-Tz2/Nb** | 7.69x10^5^ | 0.3601 | 2.13x10^6^ | 0.99871 |
| **1 mM SQ – 3 mM PEG-Tz1/Nb** | 2.52x10^5^ | 0.3154 | 8.05x10^5^ | 0.99654 |
| **2 mM SQ – 3 mM PEG-Tz1/Nb** | 6.44x10^4^ | 0.3437 | 1.87x10^5^ | 0.99470 |
| **3 mM SQ – 3 mM PEG-Tz1/Nb** | 3.85x10^4^ | 0.341 | 1.13x10^5^ | 0.99820 |
| **4 mM SQ – 3 mM PEG-Tz1/Nb** | 8.41x10^4^ | 0.2646 | 3.36x10^5^ | 0.99544 |
| **5 mM SQ – 3 mM PEG-Tz1/Nb** | 1.48x10^4^ | 0.3007 | 5.00x10^4^ | 0.99496 |
| **1 mM SQ – 3 mM PEG-Tz2/Nb** | 2.33x10^5^ | 0.3505 | 6.63x10^5^ | 0.99751 |
| **2 mM SQ – 3 mM PEG-Tz2/Nb** | 1.28x10^5^ | 0.3001 | 4.33x10^5^ | 0.99499 |
| **3 mM SQ – 3 mM PEG-Tz2/Nb** | 7.08x10^4^ | 0.2721 | 2.72x10^5^ | 0.99477 |
| **4 mM SQ – 3 mM PEG-Tz2/Nb** | 2.91x10^4^ | 0.2506 | 1.26x10^5^ | 0.99232 |
| **5 mM SQ – 3 mM PEG-Tz2/Nb** | 9.07x10^3^ | 0.2611 | 3.69x10^4^ | 0.99271 |


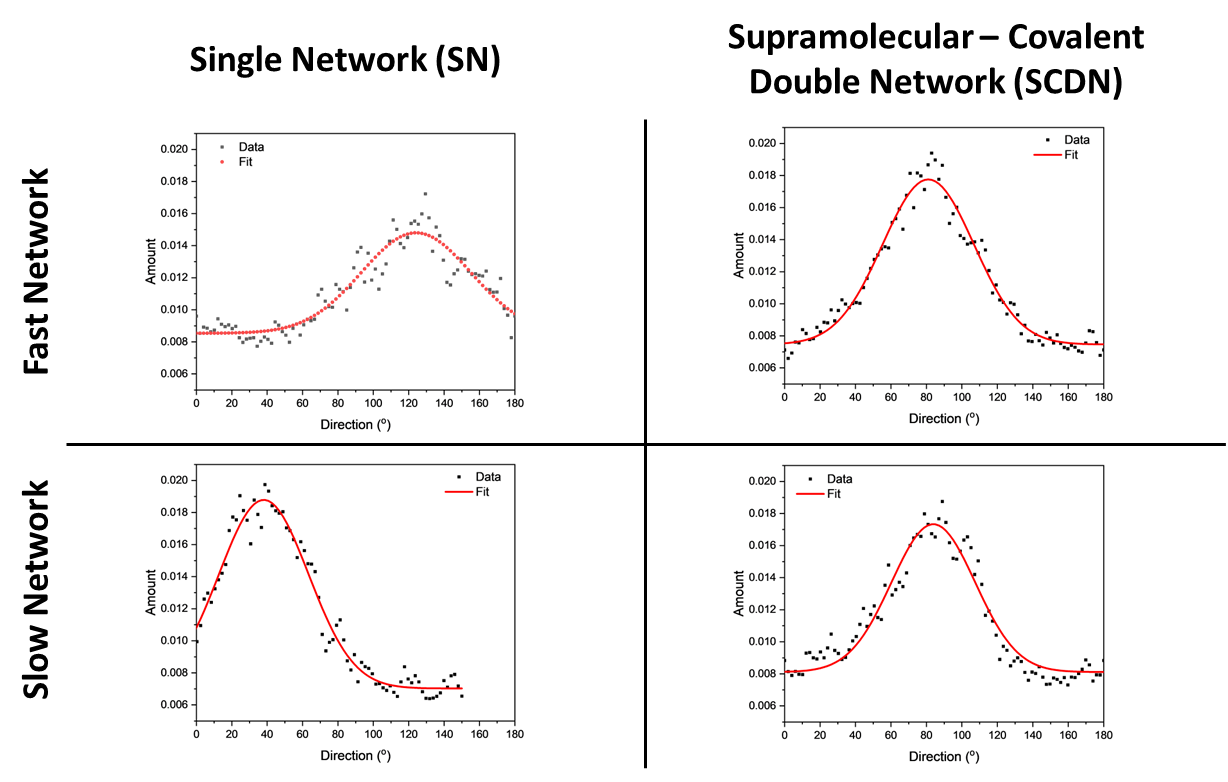


**Figure S5**. Bulk network orientation and directionality as determined from cryo-SEM images by processing using the Directionality plug-in in the Fiji software.

**Table S2.** Cryo-SEM directionality analysis of hydrogel samples.

| **Sample** | **Direction (deg)** | **Dispersion (deg)** | **Amount** | **Goodness of fit** |
| --- | --- | --- | --- | --- |
| **SN – Fast Network** | 124.17 | 30.88 | 0.73 | 0.87 |
| **DN - Fast Network** | 81.01 | 25.74 | 0.7 | 0.97 |
| **SN - Slow Network** | 38.03 | 25.31 | 0.7 | 0.97 |
| **DN - Slow Network** | 83.88 | 23.62 | 0.63 | 0.94 |


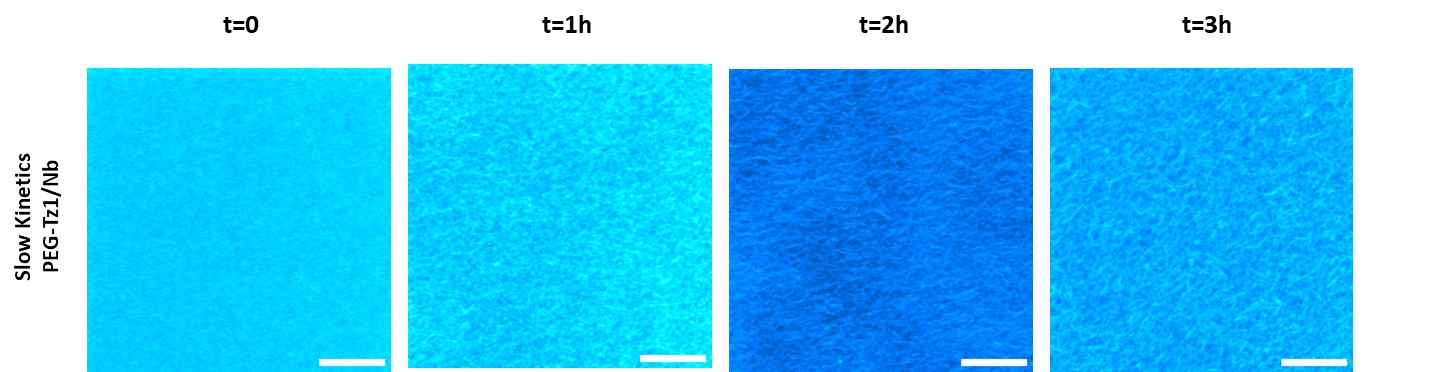


**Figure S6.** Raw micrograph data of time-dependent CLSM measurements of the slow SCDN hydrogel (4 mM **SQ** and 3 mM **PEG-Tz1/Nb**). Scale bar: 10 μm.


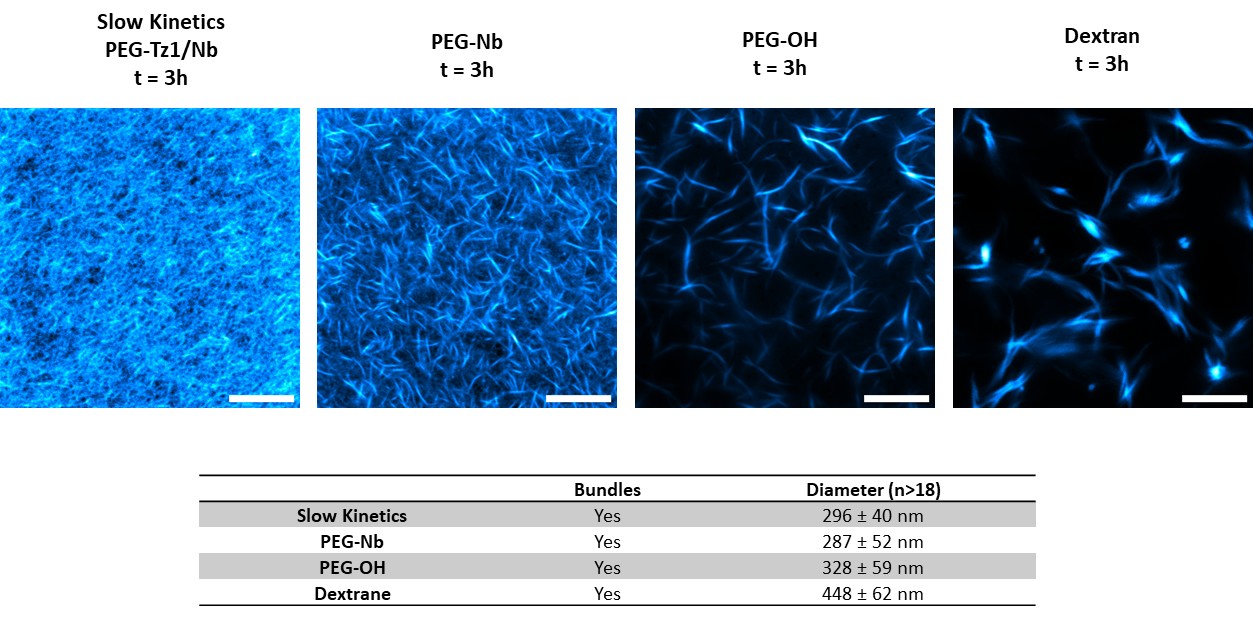


**Figure S7.** Deconvoluted confocal micrographs used for quantification of bundle thickness. The total macromolecular crowder concentrations are 3 mM for PEGs and 0.4 mM for dextran. Scale bar: 10 μm.


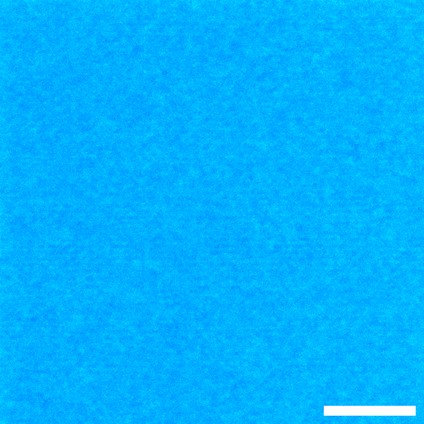


**Figure S8.** CLSM micrograph of **SQ** (4 mM). Scale bar: 10 μm.


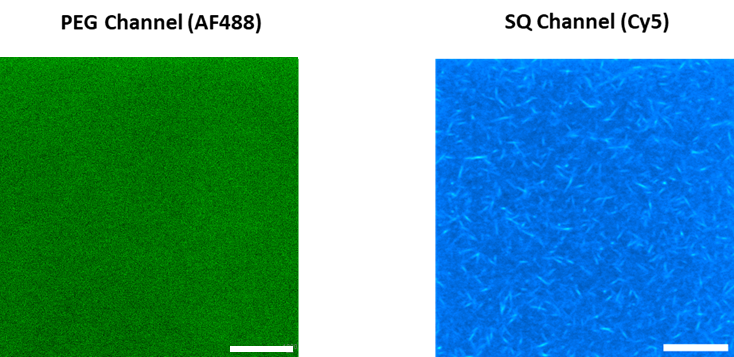


**Figure S9.** CLSM micrographs of 3 mM **PEGNb** labeled with **AF488** Tetrazine (5-Isomer) and 4 mM **SQ** with **SQ-Cy5**. Scale bar: 10 μm.


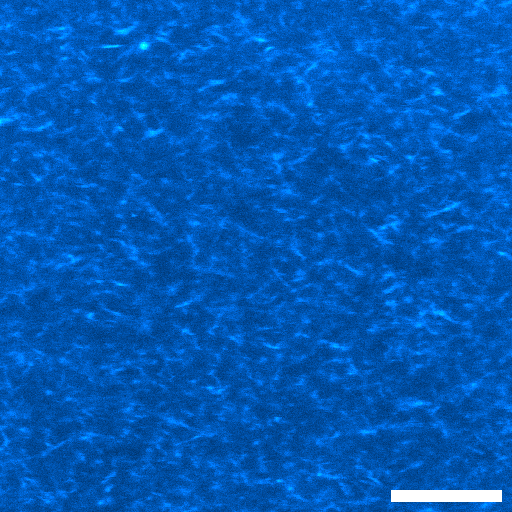


**Figure S10.** CLSM micrograph of mixture of 0.4 mM **SQ** and 5 mM **PEG-Nb**, showing the formation of bundles. Scale bar: 10 μm.


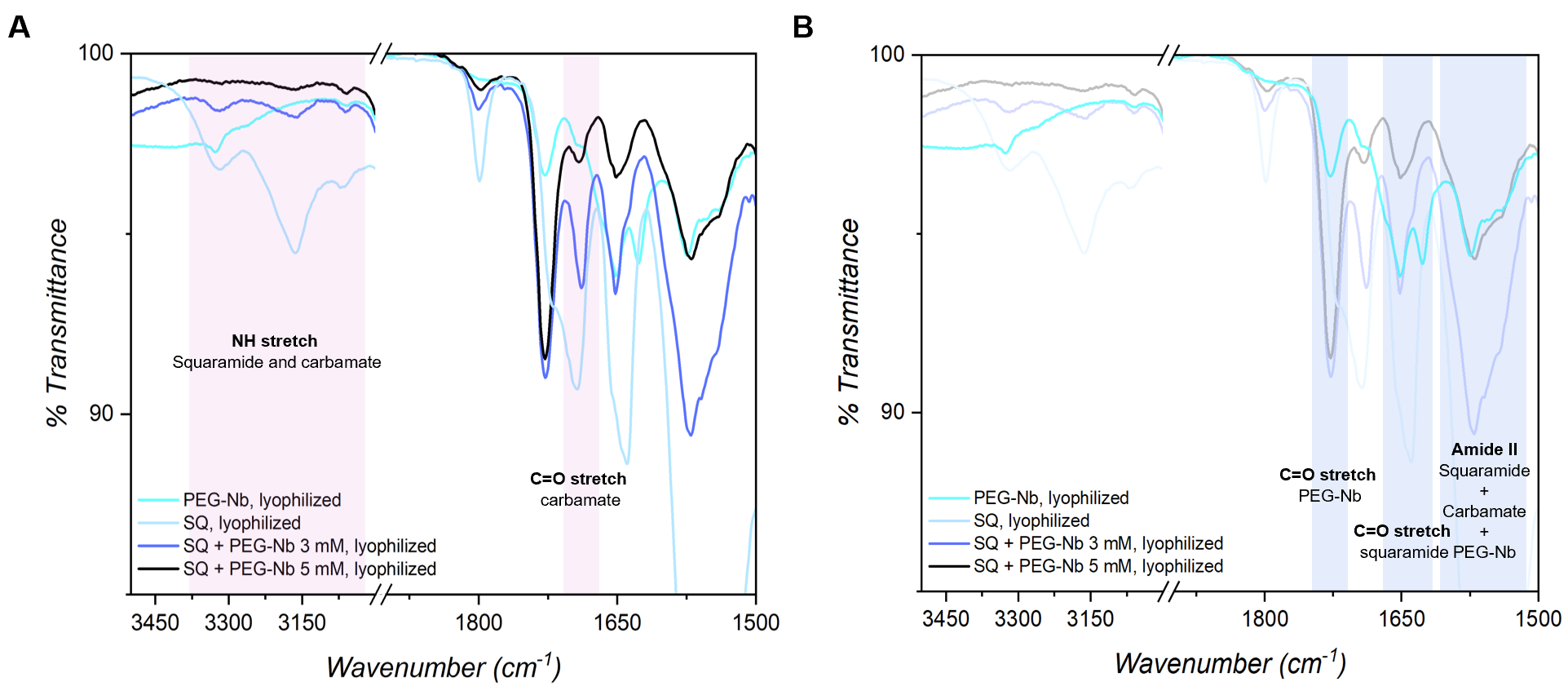


**Figure S11.** FT-IR spectra of lyophilized samples of **SQ**, **PEG-Nb** and 4 mM **SQ** and **PEG-Nb** mixtures (3 mM and 5 mM) highlighting the regions where the **PEG-Nb** is minor (A) and substantial (B).

# 3. References

(1) Tong, C.; Liu, T.; Saez Talens, V.; Noteborn, W. E. M.; Sharp, T. H.; Hendrix, M. M. R. M.; Voets, I. K.; Mummery, C. L.; Orlova, V. V.; Kieltyka, R. E. Squaramide-Based Supramolecular Materials for Three-Dimensional Cell Culture of Human Induced Pluripotent Stem Cells and Their Derivatives. *Biomacromolecules* **2018**, *19*, 4) 1091–1099.

(2) Wu, J. H.; Jia, Q. The Heterogeneous Energy Landscape Expression of KWW Relaxation. *Sci. Rep.* **2016**, *6*, 1, 1–10.

(3) Kirshner, H.; Aguet, F.; Sage, D.; Unser, M. 3-D PSF Fitting for Fluorescence Microscopy: Implementation and Localization Application. *J. Microsc.* **2013**, *249*, 1, 13–25.

(4) Sage, D.; Donati, L.; Soulez, F.; Fortun, D.; Schmit, G.; Seitz, A.; Guiet, R.; Vonesch, C.; Unser, M. DeconvolutionLab2: An Open-Source Software for Deconvolution Microscopy. *Methods* **2017**, *115*, 28–41.

(5) Ma, Y.; Zhou, Y.; Long, J.; Sun, Q.; Luo, Z.; Wang, W.; Hou, T.; Yin, L.; Zhao, L.; Peng, J.; Ding, Y. A High-Efficiency Bioorthogonal Tumor-Membrane Reactor for In Situ Selective and Sustained Prodrug Activation. *Angew. Chem. Int. Ed.* **2024**, *63*, 10, e202318372.

(6) Poulie, C. B. M.; Sporer, E.; Hvass, L.; Jørgensen, J. T.; Kempen, P. J.; Lopes van den Broek, S. I.; Shalgunov, V.; Kjaer, A.; Jensen, A. I.; Herth, M. M. Bioorthogonal Click of Colloidal Gold Nanoparticles to Antibodies In Vivo. *Chem. Eur. J.* **2022**, *28*, 61, e202201847.

(7) Tong, C.; Wondergem, J. A. J.; Van Den Brink, M.; Kwakernaak, M. C.; Chen, Y.; Hendrix, M. M. R. M.; Voets, I. K.; Danen, E. H. J.; Le Dévédec, S.; Heinrich, D.; Kieltyka, R. E. Spatial and Temporal Modulation of Cell Instructive Cues in a Filamentous Supramolecular Biomaterial. *ACS Appl. Mater. Interfaces* **2022**, *14*, 15, 17042–17054.
